# Health trainer-led motivational intervention plus usual care for people under community supervision compared with usual care alone: a study protocol for a parallel-group pilot randomised controlled trial (STRENGTHEN)

**DOI:** 10.1136/bmjopen-2018-023123

**Published:** 2018-06-04

**Authors:** Tom P Thompson, Lynne Callaghan, Emma Hazeldine, Cath Quinn, Samantha Walker, Richard Byng, Gary Wallace, Siobhan Creanor, Colin Green, Annie Hawton, Jill Annison, Julia Sinclair, Jane Senior, Adrian H Taylor

**Affiliations:** 1 Plymouth University Peninsula Schools of Medicine and Dentistry, Plymouth, UK; 2 Plymouth City Council, Plymouth, UK; 3 University of Exeter Medical School, Exeter, UK; 4 School of Law, Criminology, and Government (Faculty of Business), Plymouth University, Plymouth, UK; 5 University of Southampton, Southampton, UK; 6 Division of Psychology & Mental Health, University of Manchester, Manchester, UK

**Keywords:** offenders, community supervision, lifestyle support, health behaviour change, mental wellbeing, complex intervention

## Abstract

**Introduction:**

People with experience of the criminal justice system typically have worse physical and mental health, lower levels of mental well-being and have less healthy lifestyles than the general population. Health trainers have worked with offenders in the community to provide support for lifestyle change, enhance mental well-being and signpost to appropriate services. There has been no rigorous evaluation of the effectiveness and cost-effectiveness of providing such community support. This study aims to determine the feasibility and acceptability of conducting a randomised trial and delivering a health trainer intervention to people receiving community supervision in the UK.

**Methods and analysis:**

A multicentre, parallel, two-group randomised controlled trial recruiting 120 participants with 1:1 individual allocation to receive support from a health trainer and usual care or usual care alone, with mixed methods process evaluation. Participants receive community supervision from an offender manager in either a Community Rehabilitation Company or the National Probation Service. If they have served a custodial sentence, then they have to have been released for at least 2 months. The supervision period must have at least 7 months left at recruitment. Participants are interested in receiving support to change diet, physical activity, alcohol use and smoking and/or improve mental well-being. The primary outcome is mental well-being with secondary outcomes related to smoking, physical activity, alcohol consumption and diet. The primary outcome will inform sample size calculations for a definitive trial.

**Ethics and dissemination:**

The study has been approved by the Health and Care Research Wales Ethics Committee (REC reference 16/WA/0171). Dissemination will include publication of the intervention development process and findings for the stated outcomes, parallel process evaluation and economic evaluation in peer-reviewed journals. Results will also be disseminated to stakeholders and trial participants.

**Trial registration numbers:**

ISRCTN80475744; Pre-results.

**Strengths and limitations of this study**

- The pilot trial has developed comprehensive methods for a rare study involving offenders under community supervision across two geographical sites.
- The pilot trial employs a tailored health trainer intervention involving one-to-one support to support clients with changes in four health behaviours and well-being, which are often overlooked in the target population.
- The trial’s development involved extensive public and patient involvement to maximise acceptability and feasibility.
- The findings will inform if the progression rules are met and whether there is a case for extending the study into a definitive trial.
- The study is not powered to detect changes in the quantitative outcomes but instead aims to assess acceptability and feasibility of the trial methods and intervention.

## Background

People with experience of the criminal justice system (CJS) have greater physical and mental healthcare needs, lower psychological well-being^1^ and experience significant problems in accessing health and social care services^2^ compared with the general population in the UK. Services for those under community supervision with multimorbidities are often fragmented.^3^ A lack of trust in health services and health professionals (eg, in primary care) causes many offenders to avoid seeking medical help despite a high prevalence of emotional problems.^4^

Unhealthy behaviours such as problematic alcohol use and smoking are much higher in the offender population than the general population.^5^ For example, 60%–80% of the offender population report problematic alcohol use compared with 20%–30% in the general population and around 80% of offenders smoke compared with just under 20% in the general population.^6^ Both these behaviours (often coexisting) lead to several health problems, and possibly lower levels of mental well-being, through a number of plausible processes (eg, economic, social and psychological). Substance misuse is also prevalent, and services and treatment pathways for offenders with heroin (opiate) use disorders are well established^7^ in contrast to those with alcohol and tobacco use disorders.

The Government’s 2004 white paper ‘Choosing health: making healthy choices easier’^8^ introduced a new workforce called health trainers (HTs), often drawn from the communities in which they operate. HTs’ main role is to provide one-to-one support to people in disadvantaged areas to facilitate health behaviour change. A handbook for HTs was developed in 2008 outlining the approach and evidence-based techniques (eg, goal setting, self-monitoring and creating action plans) that HTs can use to help people change their behaviour.^9^ The core work of HTs includes the support of behaviour change such as healthy eating, stopping/reducing smoking, increasing physical activity and reducing alcohol consumption. Their work has been positively rated, but there is still a lack of robust evaluation.^10^

Our rapid review of published and grey literature, and contact with probation services leads, revealed that the scope of HTs has been extended to prison and probation settings with promising findings,^11^ especially when the HT has personal experience of the CJS. While HTs have typically focused on supporting health behaviour change, there is increasing interest in their role being extended to facilitate improvements in mental well-being. Evaluative evidence suggests where enhancing mental well-being has been the main focus of working with a HT, individuals within the CJS are more likely to attain their planned goals.^11^ In parallel work, a screening and brief intervention for reducing alcohol use in individuals in the criminal justice settings^12–14^ indicated no additional benefit in comparison with feedback on screening and a client information sheet,^15^ suggesting a more client-centred intervention with longer engagement may be needed. A recent systematic review^16^ identified 95 randomised trials involving offenders both in and out of prison (42 studies based in the community) that aimed to assess the effects of various interventions on improving health outcomes. Fifty-nine studies suggested that the intervention led to improvements in mental health, substance use, infectious disease outcomes or modified health service use. However, 91 of the studies were assessed as having an unclear or high risk of bias and the review highlighted the lack of high-quality rigorous research with a population that is comparatively under researched. Further rigorous research is therefore needed to evaluate the effectiveness and cost-effectiveness of an HT-led intervention aimed at improving mental well-being and health behaviour among people under community supervision and to understand the change processes involved.

The recent reorganisation of community supervision as part of the ‘Transforming Rehabilitation’ agenda created Community Rehabilitation Companies (CRCs) and the National Probation Service (NPS). The reforms included providing supervision to people released from prison with sentences under 1 year (ie, people who were previously unsupported). This creates a new opportunity to work with this part of the population while they are still under supervision. Providing HT support within this context could improve engagement with existing health promotion services,^17^ stimulate greater ownership and control over health behaviour change and involvement in activities to foster enhanced mental well-being.^18^

There has been increasing interest in subjective mental well-being, as a more holistic concept than just an absence of mental illness. The following five behaviours (collectively known as ‘The Five Ways to Wellbeing (5WWB)) to increase mental capacity and well-being were recommended in the Foresight Report^18^: connect with others; be physically active; take notice of things around you; keep learning; and give.

Mental well-being potentially impacts on physical health (eg, hypertension and heart disease) and mental health (eg, depression, self-harm and substance misuse); health behaviours (eg, smoking, alcohol, physical activity and diet); employment and productivity; crime; and society in other ways.^18^ While the role of exercise for improving mental well-being is clear, changing other specific health-related behaviours such as smoking may also improve subjective feelings of mental well-being for some individuals. Individuals’ patterns of current behaviour, motivation to change and potential benefits will be idiosyncratic and require a personal analysis.^18^ Assessing the benefit of health promotion interventions is rarely easy, and mental well-being poses particular problems. One method of assessing subjective mental well-being is through the Warwick and Edinburgh Mental Wellbeing Scale (WEMWBS). The WEMWBS captures the two perspectives of mental well-being: (1) the subjective experience of happiness (affect) and life satisfaction (the hedonic perspective); and (2) positive psychological functioning, good relationships with others and self-realisation (the eudemonic perspective). The latter, based on Self-Determination Theory, includes the capacity for self-development, positive relations with others, autonomy, self-acceptance and competence^19^ and, therefore, the potential to positively enhance further health promoting behaviours.

The WEMWBS has been widely used at a population level to assess mental well-being, as well as with individuals in specific groups.^20–22^ Original data obtained from the Scottish Prisoner Service showed a mean (SD) WEMWBS score of 43.2 (12.3) (range 14–70), compared with a general population score of 51.6 (8.71) for England^22^ and 49.9 (8.5) for Scotland.^23^ Lower scores are associated with smoking, lower consumption of fruit and vegetables, high alcohol intake and lower socioeconomic status.^24^

People who receive community supervision from the new NPS and CRC services are particularly suitable for a high intensity health promotion intervention for four reasons: (1) they are often excluded from ‘usual’ healthcare and health and wellbeing-promoting interventions due to a combination of access arrangements, lifestyle factors and distrust of services; (2) they often have low levels of mental well-being and poor health-related behaviours and thus the gains of the proposed intervention are potentially high; (3) while under supervision, and therefore in a period of sustained mandated contact with a service, there is an opportunity to both engage such individuals in an intervention and capture follow-up data within the context of a rigorous evaluation; and (4) being subject to justice supervision can often be a time when individuals wish to improve their life circumstances, particularly towards the start of sentences or transition into the community from prison.

The proposed research will develop and test the feasibility and acceptability of a client-centred intervention for individuals receiving community supervision aimed at improving mental well-being and other secondary outcomes and also the acceptability and feasibility of the methods involved in a randomised controlled trial (RCT). The pilot trial and parallel process evaluation, described here, will further test our assumptions, the intervention, and establish a framework for estimating cost-effectiveness. This protocol paper describes the methods for a pilot RCT with parallel process evaluation. For the full protocol, see online supplementary file.

10.1136/bmjopen-2018-023123.supp1 Supplementary file 1

### Aims and objectives

The aim of the pilot trial is to explore uncertainties about the acceptability and feasibility of the trial methods and intervention prior to progression to a definitive trial.

## Methods

This protocol is informed by Standard Protocol Items: Recommendations for Interventional Trials^25^ guidance for the reporting of clinical trial protocols.

### Study design

This study employs a parallel two-group randomised pilot trial with 1:1 individual participant randomisation to either the STRENGTHEN intervention plus standard care (intervention) or standard care alone (control) with a parallel process evaluation.

Participants are being recruited through CRCs in the Southwest and Northwest of the England, and through the NPS in the southwest only. CRCs manage cases in the community who are categorised as presenting low to medium risk of serious harm, and the NPS manage cases who present a high risk of serious harm. Participants are only being recruited through the NPS at one site to test the feasibility and acceptability of recruitment and engagement of those classified as presenting a high risk of serious harm.

### Participant inclusion criteria

Participants must satisfy the following criteria to be enrolled in the study: males and females aged 18 years or older; receiving community supervision; for prison releases, have been in the community for at least 2 months following any custodial sentence; have a minimum of 7 months left to serve of community sentence/supervision; be willing and able to receive support to improve in one or more of the four target health behaviours and/or improve mental well-being; be willing and able to take part in a pilot RCT with follow-up assessments at 3 and 6 months; and be residing within the geographical areas of the study.

### Participant exclusion criteria

Participants who meet any of the following criteria at the time of identification and screening will be excluded from study participation: those who present a serious risk of harm to the researchers or intervention practitioners; those unable to provide informed consent; and those with disrupted lives who may find it difficult from the outset to engage in the intervention and follow-up assessments. Potential participants who are not able to consent when originally approached, but who may regain this capacity (eg, due to change in intoxication), will be given a further chance to participate.

### Recruitment settings, procedures and initial approach

There are two participant identification pathways: (1) via NPS or CRC; and (2) via community organisations. Recruitment will take place in community organisations as an attempt to reach those who may not engage regularly with the CRC or NPC.

Potential participants are identified in partnership with the CRCs and NPS. Decisions whether to include someone, based on their level of risk, will be taken by the research team at each site in conjunction with local services if needed.

1. A single point of access (SPOA) administrator has been identified for both the CRCs and the NPS. The SPOA administrator identifies potential participants from the nDelius record system for all services. Offender managers (OMs) of identified individuals are consulted for screening of inclusion/exclusion criteria and assessment of risk. On receipt of clearance to approach potential participants, OMs ask clients if they agree to speak to the site researcher either at their next scheduled appointment or via the telephone (depending on the current mode of contact between the OM and potential participant within their community supervision). On receiving verbal agreement to approach, the OM facilitates the researcher making the initial approach either in person, following the individual’s routine appointment at CRC/NPS or via the telephone. All potential participants are offered the opportunity to meet the researcher for the initial appointment in a meeting space at CRC/NPS offices. Identification of participants through community organisations involve staff initially approaching potential participants to invite them to talk to a researcher about the study. On receiving verbal agreement to approach, the researcher will make a time and date for a meeting. The researcher will explain the study and provide the opportunity to ask questions. If the individual expresses an interest in taking part in the study, the researcher progresses with the consent process.
2. Community organisations including drug and alcohol rehabilitation centres, hostels and day centres will also support initial identification of potential participants. The consent form for participants who are identified through community organisations requests consent for the researcher to make contact with the participant’s OM in order to check that they meet the criteria for participation in the study. Following positive assessment by the OM, the researcher will contact the participant to make a time to conduct the baseline data collection. If the OM assesses the participant as not meeting the criteria for inclusion in the study, the researcher will make a time to explain why the participant is unable to take part in the study.

### Screening, baseline and informed consent

Following the initial approach, if a potential participant expresses an interest in taking part in the study, a meeting is arranged between the researcher and the potential participant where the researcher explains the project in more detail. This meeting may take place immediately after the initial approach, but the potential participant can take longer to consider if they want to take part if necessary.

The researcher reads and explains the information in the participant information sheet (PIS), including time burden, at the initial meeting, showing sensitivity to the high levels of often undeclared literacy difficulties in this population. The researcher places particular emphasis on ensuring that the potential participant fully understands the concept and implications of randomisation, the voluntary nature of the research and their right to withdraw at any time without detriment to their care or legal rights. Confidentiality arrangements (including reasons for breaching confidentiality) and data protection are also presented.

Having had the opportunity to discuss their involvement in the study and ask questions about it, potential participants are asked if they are:

- Willing and able to receive support to improve one or more of the four target health behaviours and/or improve mental well-being if randomised to the intervention.
- Willing and able to take part in a pilot RCT with follow-up assessments at 3 and 6 months.

If a potential participant is unwilling or unable to proceed, they are thanked for their time and contribution, reminded that there are no negative consequences of not taking part, and their involvement will end. If a potential participant is both willing and able to proceed to the trial, the consent form is explained to them before both the participant and the researcher sign two copies (one retained by the participant and one by the researcher). The researcher continues with the baseline data collection during this same visit/meeting, checking that the participant is happy to proceed or makes a further appointment for data collection. In the unusual circumstance that the baseline data collection occurs more than 2 weeks after initial screening, a rescreening will take place prior to baseline data collection.

The researcher delivers the baseline data collection assessment using the narrative conversational format developed in our previous studies.^7^ The questions from the WEMWBS (the primary outcome) are read out to participants in a precise and consistent manner should the participant prefer/require this rather than completing it themselves (method of completion is recorded). Questions from other measures are incorporated into a specially constructed flexible script that avoids duplication of subject matter to minimise disengagement or irritability. As per the consent process, individuals who lack capacity on a particular day will be given additional opportunities to complete the baseline data collection assessment, before being deemed to be ineligible to continue participation in the study. This is a particularly important allowance when the population of interest often live challenging lives with competing priorities.

## Confidentiality

### Randomisation

Allocation to intervention or control group uses minimisation, with a random element, to ensure balance between treatment arms with respect to age, gender and recruitment site. Recruitment site is determined by a combination of geographic region and the service type: (1) Northwest CRC; (2) Southwest CRC; and (3) Southwest NPS. Allocation is achieved by means of a web-based system created and maintained by the Peninsula Clinical Trials Unit.

Once the participant has completed the screening interview and baseline data collection assessment, the researcher/administrator accesses the randomisation website using a unique username and password. The website requires entry of the study site, participant’s initials, participant’ s date of birth and gender, before returning the participant’s unique randomisation number and allocation (intervention or control) to the trial administrator via email. The website confirms that the allocation process has been successful but does not display the participant’s allocated group at the point of entry to maintain blinding of the research assistants (RAs). The first participant was randomised on 18 October 2016.

## Sample size

A formal sample size calculation based on considerations of power is not appropriate; this pilot study is not powered to detect between-group clinically meaningful differences in a primary outcome. The aim is to provide robust estimates of the likely rates of recruitment and follow-up, as well as to provide estimates of the variability of the proposed primary and secondary outcomes to inform sample size calculations for the planned definitive trial.

When data from a pilot study are required to estimate the SD of a continuous outcome, to maximise efficiency in terms of the total sample size across pilot and main trials, the recommendation is that a two-group pilot study should have follow-up data from at least 70 participants (ie, 35 per group).^26^ When considering binary outcomes, a total of at least 120 participants is recommended.^26^ For the pilot RCT (phase 2), we believe that over 3 months, and across the two sites, we will be able to approach around 330 potential participants. We aim to recruit at least 120 participants across the two geographical regions (60 per region).

### Treatments

#### Control arm

Individuals in the control group will receive treatment as usual, which will include support from the CJS and any other third sector or healthcare organisations in the standard way. For each site, we will identify what support participants would normally receive while working with the NPS and CRCs, and this will be documented, updated and maintained. Participants in both arms of the study will have access to all local services as usual.

#### Intervention arm

Through original research and literature reviews, we have developed an extensive understanding of what are likely to be the effective components of an intervention targeted at health behaviours and improvement of health and mental well-being in this population. A clear starting point logic model (which will be presented in more detail elsewhere) of intervention components and aims underpins the intervention, based on the HT role in a previous trial of smoking cessation in disadvantaged groups^27^ and the development of a collaborative care model for prison leavers with multiple health problems.^28^ The intervention aims to enhance people’s mental well-being and improve their health-related behaviours.

The HT role has been adapted for specific populations, including offenders^11^ and smokers,^27^ with early signs that the support is acceptable and feasible. However, further intervention development and piloting was required to integrate a focus on promoting mental well-being and multiple health behaviour changes in offenders in the new NPS/CRCs context and to understand the interactions between mental well-being and health behaviour changes. These uncertainties have been explored, and reduced, in a formative process evaluation working with the peer researchers, people with lived experience of the CJS.

A training package was delivered to the HTs on the project focusing on the core competencies of an HT as outlined in the HT handbook^9^ with training in the 5WWB. During the manualisation phase, the HT handbook was adapted to incorporate the principles of 5WWB and tailored for working with the target population.

The key components of the intervention are:

1. An HT is available for one-to-one sessions over 14 weeks, in face-to-face or telephone format (frequency and length of sessions is negotiated with each participant). We expect an average of 4–6 sessions (with greatest results being achieved up to six sessions with diminishing returns beyond that^29^). The face-to-face intervention sessions take place in a variety of settings, including probation services and other local community locations. Initial engagement and proactive follow-up is based on our previous offender research.
2. An initial invitation to engage with the HT is described as an ‘open and flexible’ opportunity to receive support for one or more of the target health behaviours and/or improving overall health and mental well-being through other activities including connecting, keeping learning, being active, taking notice and giving (ie, the 5WWB).
3. HTs are trained to help participants understand the inter-relationship between health behaviours such as smoking, alcohol use, diet, physical activity and their relationship to mental well-being and other positive and negative behaviours, including substance use. Each participant develops a personal plan based on individual behaviour change goals and motivation to improve mental well-being. Some offenders will have positive perceived mental well-being but engage in negative behaviours, while others will be as concerned about emotional distress. The intervention is intended to be flexible enough to support both these extremes.
4. The support is described as ‘open’ to reflect the planned underpinning and overlapping influence of Self-Determination Theory and the client-centred principles of Motivational Interviewing.^30^ HTs avoid giving ‘advice’ and empower clients to confirm the desire for change and develop self-regulatory skills such as self-monitoring, setting action plans and reviewing progress. The intervention is tailored and led by the participants’ needs.
5. The HT, informed by the 5WWB, helps clients to build positive behaviours (eg, initiating and maintaining activities (physical, creative and so on) and find opportunities for gaining core human needs (ie, sense of competence, autonomy and relatedness), as well as learn and notice, to enhance mental well-being.
6. Any reductions in alcohol consumption (as units per week, alcohol-free days or avoidance of trigger events^31^), smoking (using different strategies^27 32^) and increases in physical activity and healthy eating are supported, with the aim to build confidence to meet guidelines for safe alcohol consumption, to quit/reduce smoking, engage in daily/weekly physical activity and healthy eating.
7. Participants are actively supported to gain help from friends and family, link with other community resources (parks and leisure centres) and services (eg, Stop Smoking Services and Drug and Alcohol Treatment Service) as a part of achieving their personal plan, exploring options for continued support after the intervention as appropriate. We have found signposting alone to be insufficient with this population.^27^

## Blinding

Blinding of the researchers is being tested for feasibility to see whether it would be possible in the definitive trial. Researchers record instances where they believe they have been unblinded in any way.

### Outcome measurement

#### Feasibility outcomes

The study aims to collect data on the following acceptability and feasibility outcomes: proportion of eligible participants; recruitment rates; rates of attrition and loss to follow-up; completion and completeness of data collection; estimates of the distribution of outcome measures; acceptability of intervention to participants; and acceptability of study participation to participants.

#### Assessments

Data are collected in the following areas as proposed outcome measures to be used in a future definitive trial and to assist with predicting SD size for future sample size calculations: subjective mental well-being (WEMWBS)^20 21 33 34^; self-reported smoking (number of cigarettes smoked per day)^27^; Fagerström Test for Cigarette Dependence^35^; alcohol use Alcohol Use Disorder Identification test (AUDIT-C)^36^; diet (Dietary Instrument for Nutrition Education)^37^; physical activity (7-day recall of physical activity)^38^; substance use Treatment Outcomes Profile (TOPS)^39^; confidence, importance, access to social support, action planning and self-monitoring measures relating to the four health behaviours; health-related quality of life (EQ-5D-5L and SF-36); and health, social care, criminal justice and voluntary sector resource use (see table 1).

**Table 1:** SPIRIT table study schedule

		Baseline assessment				
		Screening	Baseline data	Allocation		
Timepoint		***t_1_***	***t_1_***		***+3 months*** ***T_2_***	***+6 months*** ***T_3_***
Enrolment:						
Eligibility screen		X				
Informed consent		X				
Allocation				X		
Interventions:						
Intervention group:	Strengthen intervention			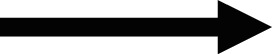		
	Usual care	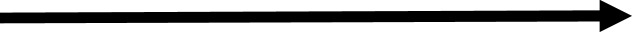				
Control group:	Usual care	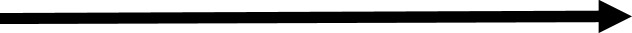				
Assessments:						
Demographics			X			
WEMWBS			X		X	X
AUDIT-C			X		X	X
DINE			X		X	X
7-day PA recall			X		X	X
Self-reported smoking			X		X	X
FTCD			X		X	X
Importance, confidence, social support, action planning and self-monitoring			X		X	X
Treatment Outcomes Profile			X		X	X
EQ-5D-5L Questionnaire			X		X	X
SF-36			X		X	X
Resource use questionnaire			X		X	X
Safety monitoring:						
Adverse event reporting						

AUDIT-C, Alcohol Use Disorder Identification Test; DINE, Dietary Instrument for Nutrition Education; FTCD, Fagerström Test for Cigarette Dependence; PA, physical activity; SF-36, 36-Item Short Form Survey; WEMWBS, Warwick and Edinburgh Mental Wellbeing Scale.

## Data collection

### Process evaluation

A parallel process evaluation is taking place alongside the pilot trial.

#### Aims

The aims of the process evaluation are: (1) to assess whether the intervention is being delivered as per manual and training; (2) to ascertain components of the intervention that are critical to delivery; (3) to explore reasons for divergence from delivery of intervention as manualised; (4) to understand when context is moderating delivery; (5) to understand the experience and motivation of participants in the control arm of the pilot to maximise retention in a full trial; (6) to explore reasons for declining to participate in the trial; (7) to explore reasons for disengaging in the intervention before an agreed end; and (8) to understand, from a participant perspective, the benefits and disadvantages of taking part in the intervention.

#### Data collection

The mixed methods data collection will include:

Face-to-face semistructured interviews will be conducted with:

- STRENGTHEN HTs (n=6) across both geographical regions.
- CRC and NPS staff (n=6) across both geographic regions.
- Participants who disengaged before an agreed end (up to 6).
- Participants randomised to the intervention arm of the pilot (high and low levels of engagement) (n=6).
- Participants randomised to the control arm of the pilot (n=6).

All interviews will be digitally audio-recorded and transcribed verbatim.

We will also collect:

- Field notes, written by RAs, on potential participants reasons for declining to participate in the study, while being sensitive to their rights to decline further participation without providing a reason.
- Digital audio recordings of HT sessions (n=20). Consent to record sessions will be sought at the start of the intervention and reconfirmed at each session prior to recording.
- HT session report forms. HTs will keep a record of each session, including information on: date, location, duration, type (face to face or by telephone), subsidy use, primary goals of the participant, goals met (if applicable) and any difficulties encountered for discussion in supervision.

#### Analysis

Intervention fidelity will be assessed through the scoring of audio recordings of HT sessions against a developed list of six key intervention processes: (1) active participant involvement; (2) motivation building for changing a behaviour and improving mental well-being; (3) set goals and discuss strategies to make changes; (4) review efforts to make changes/problem solving; (5) integration of concepts;and (6) engaging social support. These will be scored on two domains: practitioner adherence to the protocol and competence of delivery.

Quantitative data will be summarised descriptively, with CIs as appropriate. Any factors that are identified as possibly contributing to participants’ intervention engagement and trial recruitment and retention will be explored in more detail in the qualitative data. Data from the qualitative sources (eg, interviews and audio recordings) will be synthesised into a Framework Analysis grid supported by NVivo 10 software.^40^ The deductively driven components of the framework analysis will explore the feasibility and acceptability of the intervention and the research data collection techniques. Quantitative and qualitative data will also be compiled into case studies for a purposively selected subsample of participants to maximise understanding of how the intervention is, or is not, working for individuals.^41^ Any procedures that need to be adapted will be identified and improvements, and solutions will be identified. The size and impact of potential changes will inform a decision as to whether this is an internal or an external pilot trial prior to progression to a definitive trial.

#### Contribution

The process evaluation will contribute to the research through: (1) revision of the logic model of how we understand the intervention to work, development of the way in which we deliver the intervention and how we should optimise research data collection in a definitive trial; (2) identification of which areas of the intervention are not being delivered as intended to help plan for future training and development in a definitive trial; (3) generalisable learning about the feasibility and acceptability of trial procedures with this population; (4) the decision as to whether to progress to a full trial or not; and (5) the design of the process evaluation for a full trial.

### Statistical analysis

#### Quantitative

An initial analysis after 6-month follow-up is completed will focus on (1) recruitment and retention; and (2) adherence to the intervention:

1. A Consolidated Standards of Reporting Trials (CONSORT) diagram will provide a detailed description of numbers approached, meeting eligibility, having baseline data collected, being randomised and having follow-up data collected.
2. A descriptive analysis will report on the proportions of those randomised to the intervention and who attended two or more sessions, completed all sessions and set behaviour change goals in personal plans.

Data from screening, recruitment and follow-up logs will be used to generate realistic estimates of eligibility, recruitment, consent and follow-up rates in the study population to assess the feasibility outcomes of the study. We will also estimate completion rates for each of the proposed outcome measures at each time-point. All such estimates will be accompanied by appropriate confidence intervals to allow conservative assumptions to be made in the planning of the definitive trial. Individuals lost to follow-up will be compared with those who complete the pilot study to identify any potential bias.

It is inappropriate to use pilot study data to formally test treatment effects, therefore the statistical analyses will be of a descriptive nature.^42 43^ We will follow the CONSORT extension for reporting of pilot and feasibility studies^43–45^ and take note of the CONSORT extension for reporting of patient-reported outcomes.^46^ Descriptive statistics of the proposed primary and secondary outcomes will be produced, as appropriate for each measure for each group. Interval estimates of the potential intervention effects, relative to usual care, will be produced in the form of a 95% CI to ensure that the effect size subsequently chosen for powering the definitive trial is plausible, but no formal hypothesis testing will be undertaken of the pilot data.^42^ Analyses will be on an intention-to-treat basis.

#### Economic analysis

The pilot study will be used to estimate the resource use and costs associated with the delivery of the intervention and to develop a framework for estimating the cost-effectiveness of the STRENGTHEN intervention plus usual care, versus usual care alone, in a future economic evaluation alongside a fully powered RCT. We will develop and test economic evaluation methods for the collection of resource use data, and for estimating related costs, and pilot the collection of outcome data appropriate for economic evaluation. In a future full economic evaluation, it is anticipated that the primary perspective for analyses will be that of the National Health Service (NHS) and Social Care Services (ie, third-party payer), with a broader participant and societal perspective explored in sensitivity analyses, and this will guide the development of the methodological framework in the pilot study.

The key areas of resource use and costs associated with the delivery of the intervention will be identified (eg, HT time, training, supervision, travel and consumables), and methods will be tested for the collection of these data. This will be via within-trial participant level records of HT input (including contact time and non-contact time). Data on participant health service use, social care service use and other broader aspects of resource use will be collected using self-report (interviewer administered) questionnaires at baseline, 3-month and 6-month follow-ups. This resource use questionnaire will be developed specifically for this participant population, using the approach described for the Client Service Receipt Inventory^47^, and based on our experience of collecting resource use data in a wide range of prior studies.

A future full economic evaluation will present the cost-effectiveness analysis with the incremental cost per unit of change on the primary outcome measure (expected to be the WEMWBS). The primary economic endpoint of policy relevance will be the incremental cost per quality-adjusted life year (QALY) gained. QALYs will be estimated using participants’ data collected using the EQ-5D-5L^48^ and the recommended value set for England.^49^ Given uncertainty associated with estimating QALYs for this population, the SF-36, from which the SF-6D can be derived,^50^ will also be used to estimate QALYs in sensitivity analysis. EQ-5D-5L and SF-36 data are collected at baseline, 3-month and 6-month follow-ups, and the pilot study will assess the feasibility of use and completion rates regarding these measures.

A future economic evaluation is expected to include extrapolation from the trial outcomes to extend the trial-based cost-effectiveness analysis over the longer term, for example, using 1-year and 2-year time horizons. Such mathematical modelling would involve evidence synthesis and the use of assumptions, and the pilot study will be used to consider these issues in the context of future research. In addition, the pilot study data will be described in a cost-consequences framework, which presents costs and outcomes in a disaggregated, tabular format.^51 52^

### Patient and public involvement

Previous work with the target population, conducted by the authors, established peer researcher groups and the current study drew on these to help revise and focus the research question. In the early stages of this pilot trial, two public and patient involvement (PPI) groups (one male and one female) were established and informed the design of the pilot trial and intervention. They also advised on the time, duration and frequency of intervention contacts to ensure an acceptable level of burden. The PPI groups helped informed recruitment methods to ensure acceptability. PPI representatives form part of the trial steering committee to guide the conduct of the study.

A short report will be made available to participants at both study sites, as well as disseminated via email and social media.

## Trial status

Recruitment was originally due to cease in December 2016. Due to a delay in securing a second site along with a major restructuring of the host organisations (the NPS and the CRCs), the recruitment window was extended and the final participant was recruited on 7 December 2017. Data follow-up is ongoing and is planned to be completed in June 2018. Decisions concerning progression to a definitive trial will then take place.

## Discussion

The present pilot study aims to reduce uncertainties in acceptability and feasibility of the intervention and trial methods. The work presents a unique opportunity to explore if and how best to recruit traditionally hard-to-reach participants and follow them up for up to 6 months. A few small studies that generally lack methodological rigour have been conducted, and this study seeks to determine if more robust methods can be used and what challenges may be faced and how to overcome them. The intervention has been adapted from existing service delivery in what appears to be isolated locations. The present study involves carefully defining the intervention components and observing how participants engage in it, how the manualised intervention is delivered and received and whether there are factors that influence acceptability and feasibility.

Should the intervention, trial methods and choice of outcome measures be shown to be acceptable and feasible, and estimates of likely impact on primary and secondary outcomes can be produced with some confidence, then support to progress onto a definitive trial will be requested. If important changes are needed in either the intervention or trial methods, then it will be appropriate to make these before further progression to a definitive trial. In the first instance, we will describe the study as an internal pilot trial, and in the second instance, an external pilot trial.

### Ethics and dissemination

Dissemination will include publication of the intervention development process and findings for the stated outcomes, parallel process evaluation and economic evaluation in peer-reviewed journals. Results will also be disseminated to stakeholders and trial participants.

### National Offender Management Service (NOMS) approvals

The study has been approved by NOMS in conjunction with the NHS Research Ethics Committee (REC) procedures. It is a requirement of NOMS that all research involving participants under NPS and CRC supervision is approved through this process.

## Supplementary Material

Reviewer comments

Author's manuscript
