# Supplementary material for: Health trainer-led motivational intervention plus usual care for people under community supervision compared with usual care alone: a study protocol for a parallel-group pilot randomised controlled trial (STRENGTHEN)
Source: BMJ Open. 2018 Jun 4;8(6):e023123. doi: 10.1136/bmjopen-2018-023123 (PMC5988189; doi:10.1136/bmjopen-2018-023123)
Supplement: Supplementary file 1 [file bmjopen-2018-023123supp001.pdf]

STRENGTHEN:
Health Trainers for people receiving Community Supervision

Improving health, under community supervision, with the support of a Health Trainer: Evaluating a pilot randomised controlled trial

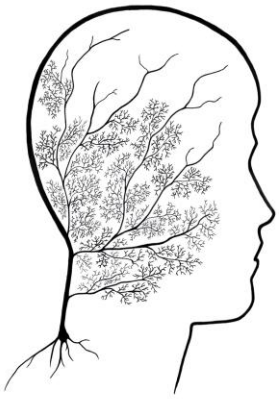

STUDY PROTOCOL

Version: 3.0
13.03.2017

REC Reference: 16/WA/0171
IRAS number: 179935
ISRCTN: 80475744
NIHR PHR: 14/54/19

Study Sponsor: Pam Baxter on behalf of Plymouth University
Chief Investigator: Professor Adrian Taylor (Plymouth University)

TABLE OF CONTENTS

TABLE OF CONTENTS 2
1 SIGNATURE PAGE 3
2 KEY CONTACT DETAILS 5
3 STUDY SUMMARY 7
4 BACKGROUND AND RATIONALE 9
5 AIMS AND OBJECTIVES 11
6 STUDY DESIGN 12
6.1 Summary 12
6.2 Setting 13
6.3 Outcome measures 13
6.3.1 Acceptability and feasibility outcomes 13
6.3.2 Secondary outcome measures/proposed outcomes measures for future definitive RCT 13
6.3.3 Economic outcomes: 13
6.4 Considerations for minimising bias 14
6.4.1 Attrition bias 14
6.4.2 Potential contamination between trial arms 15
7 STUDY PARTICIPANTS 15
7.1 Participants 15
7.1.1 Inclusion criteria 15
7.1.2 Exclusion Criteria 16
8 STRATEGIES FOR PATIENT IDENTIFICATION 16
8.1 Database search 16
8.2 Initial approach and provision of study information 16
9 STUDY SCHEDULE 17
9.1 Baseline visit 17
9.1.1 Consent Process 17
9.1.2 Baseline data collection (t_1) 19
9.1.3 Communicating allocated group to participants 19
9.2 3-month outcome measure collection (t_2) 20
9.3 6-month outcome measure collection (t_3) 20
10 MINIMISING ATTRITION 21
10.1.1 Return to prison 21
11 INTERVENTION 21
11.1 Description 21
11.2 Delivery 23

11.3 Withdrawal from intervention **24**
11.3.1 Return to prison 24
12 CONTROL GROUP 24
13 PILOT TRIAL PROCESS EVALUATION 24
14 SAFETY REPORTING 26
14.1 Definitions **26**
14.2 Reportable events **27**
14.2.1 Reporting Serious Adverse Events 27
14.3 Study Numbering **29**
14.4 Data Collection **29**
14.5 Data entry **29**
14.6 Data Confidentiality **29**
14.7 Archiving **29**
15 DATA ANALYSIS CONSIDERATIONS 30
15.1 Sample Size **30**
15.2 Statistical analysis **30**
16 ECONOMIC EVALUATION 31
17 DATA MONITORING AND QUALITY ASSURANCE 32
18 STUDY ORGANISATIONAL STRUCTURE 32
18.1 Project Management Group (PMG) **32**
18.2 Trial Steering Committee (TSC) responsibility **32**
18.3 Data Monitoring Committee (DMC) **33**
19 DIRECT ACCESS TO SOURCE DATA AND DOCUMENTS 33
20 RESEARCH GOVERNANCE 33
20.1 Sponsor **33**
20.2 Ethics and NHS approvals **33**
20.3 National Offender Management Service (NOMS) approvals **33**
21 INDEMNITY AND INSURANCE 34
22 PUBLICATION POLICY 34
23 FINANCE 34
24 REFERENCES 35
25 APPENDICES 38

Table 1: Tabulated summary of study schedule 18

1 SIGNATURE PAGE

Role	Name	Signature	Date
Chief Investigator	Prof Adrian Taylor		
Principal Investigator	Dr Jane Senior (Manchester)		
Principal Investigator	Dr Cath Quinn (Plymouth)		
Statistician	Siobhan Creanor		
Sponsor's representative	Pam Baxter		

2 KEY CONTACT DETAILS

Chief Investigator: Prof **Adrian Taylor**<br><br>Professor in Health Services Research<br><br>Room N6, ITTC Building<br>Plymouth Science Park, Derriford, Plymouth, PL6 8BX<br><br>Phone: 07952 400835<br>Email: adrian.taylor@plymouth.ac.uk	Project Manager: Dr **Lynne Callaghan**<br><br>Senior Research Fellow<br><br>N9, ITTC Building,<br>Plymouth Science Park, Derriford, Plymouth, PL6 8BX<br><br>Phone: 07807 966235<br>Email: L.Callaghan@plymouth.ac.uk
Principal Investigator (Plymouth): Dr **Cath Quinn**<br><br>Senior Research Fellow<br><br>Room N6, ITTC Building,<br>Plymouth Science Park, Derriford, Plymouth, PL6 8BX<br><br>Phone: 07817 315243<br>Email: cath.quinn@plymouth.ac.uk	Principal Investigator (Manchester): Dr **Jane Senior**<br><br>Research Manager<br><br>Room 2.317, Jean McFarlane Building<br>University of Manchester, Oxford Road, Manchester, M13 9PL<br><br>Phone: 0161 275 0730<br>Email: Jane.Senior@manchester.ac.uk
Trial Statistician: Dr **Siobhan Creanor**<br><br>Associate Professor in Clinical Trials and Medical Statistics<br><br>Room N15, ITTC Building,<br>Plymouth Science Park, Derriford, Plymouth, PL6 8BX<br><br>**Phone:**<br>**Email:** siobhan.creanor@plymouth.ac.uk	
Sponsor Representative: Ms **Pam Baxter**<br><br>Research Governance Officer<br><br>Biomedical Research – Translational & Stratified Medicine<br><br>Plymouth University<br><br>Phone: 07572 267245<br>Email: pam.baxter@plymouth.ac.uk	

LIST OF ABBREVIATIONS

5WWB	5 Ways to Wellbeing
AE	Adverse Event
AUDIT	Alcohol Use Disorders Identification Test
CI	Chief Investigator
ConSORT	Consolidated Standards of Reporting Trials
CRCs	Community Rehabilitation Companies
CRF	Case Report Form
CRN	Clinical Research Network
CSRI	Client Service Receipt Inventory
CTU	Clinical Trials Unit
CJS	Criminal Justice System
DINE	Dietary Instrument for Nutrition Education
DMC	Data Monitoring Committee
FPE	Formative Process Evaluation
GCP	Good Clinical Practice
HT	Health Trainer
ITT	Intention to Treat
NIHR	National Institute of Health Research
NOMS	National Offender Management Service
NRES	National Research Ethics Service
OM	Offender Manager
PenCTU	Peninsula Clinical Trials Unit
PPI	Patient and Public Involvement
PIS	Patient Information Sheet
QALY	Quality Adjusted Life Year
RA	Research Assistant
R&D	Research and Development
RCT	Randomised Controlled Trial
SAE	Serious Adverse Event
SD	Standard Deviation
SOP	Standard Operating Procedure
SPOA	Single Point of Access
TOP	Treatment Outcomes Profile
TSC	Trial Steering Committee
UKCRC	United Kingdom Clinical Research Collaboration
WEMWBS	Warwick and Edinburgh Mental Wellbeing Scale

3 STUDY SUMMARY

**Study Title**	Improving health, under community supervision, with the support of a Health Trainer: Developing and evaluating a pilot randomised controlled trial.
**Study Design**	A two centre parallel group pilot randomised controlled trial with parallel process evaluation.
**Study Participants**	Males and females aged 18 years and older who have at least 7 months of a community supervision order left to serve. For those recently released from prison to community supervision; resident in the community for at least two months. Willing to receive support for improving lifestyle and wellbeing.
**Intervention**	Usual care plus receipt of the STRENGTHEN Intervention consisting of up to 12 one-to-one sessions (face-to-face and by telephone) over 14 weeks with support from an enhanced Health Trainer to improve wellbeing and improve health behaviours.
**Control**	Usual care alone.
**Study duration**	24 months.
**Nº of participants**	120 participants will be randomised to either the Intervention (n=60) or Control (n=60) arm.
**Setting**	2 cities involving a total of 3 offender services: Plymouth (Community Rehabilitation Company + National Probation Service) and Manchester (Community Rehabilitation Company).
**Aims**	To develop and implement a Health Trainer-led intervention supporting health and wellbeing improvements for those under community supervision, within the context of a pilot randomised trial.
**Specific objectives**	1. To assess the acceptability and feasibility of such an intervention, alongside routine engagement with community supervision services, for the key stakeholders including Community Rehabilitation Companies (CRCs), the National Probation Service (NPS), Health Trainers and those receiving community supervision.<br><br>2. To assess the acceptability of recruitment, assessment and randomisation procedures within a pilot pragmatic randomised controlled trial of the intervention versus usual care (to be defined by service observation, but likely to be minimal).<br><br>3. To determine, from the pilot randomised controlled trial (RCT), completion rates for proposed outcome measurements to assess wellbeing (WEMWBS) and behavioural measures (e.g. self-reported alcohol consumption, smoking, diet, physical activity, substance use), and quality of life (SF36 and EQ-5D-5L) at baseline and 3- and 6-month follow-up.<br><br>4. To provide data to contribute to sample size calculations for a fully-powered RCT to primarily assess subjective wellbeing (WEMWBS) and to ensure that the effect size (intervention vs. usual care) chosen for powering the definitive trial is plausible.<br><br>5. To use two-stage, mixed methods, process evaluation to further refine and understand the acceptability and feasibility of the intervention, its delivery and the trial procedures. The findings will be used to refine the intervention and the logic model of the causal assumptions that underpin it.

	6. To estimate the resource use and costs associated with delivery of the intervention, and to pilot methods for the cost-effectiveness framework in a full trial.
**Feasibility outcomes**	• Proportion of eligible participants<br>• Recruitment rate<br>• Attrition and loss to follow-up<br>• Completion and completeness of data collection<br>• Estimates of the distribution of outcome measures<br>• Acceptability of intervention to participants<br>• Acceptability of study participation to participants
**Secondary Outcomes**	• The Warwick and Edinburgh Mental Wellbeing Scale (WEMWBS) at 6-months post-baseline.<br>• Self-reported smoking (n cigarettes smoked per day);<br>• Fagerström Test for Cigarette Dependence<br>• Alcohol use (AUDIT-C);<br>• Diet (Dietary Instrument for Nutrition Education [DINE]);<br>• Physical Activity (7-day recall physical activity questionnaire);<br>• Substance use (Treatment Outcomes Profile [TOP]);<br>• Confidence, importance, access to social support, action planning, and self-monitoring measures relating to health behaviours<br>• Health related quality of life (EQ-5D-5L, SF-36);<br>• Cost effectiveness
**Inclusion Criteria**	• Males and females;<br>• 18 years and older;<br>• At least 7 months of community supervision left to serve;<br>• For prison leavers, released a minimum of two months prior to recruitment;<br>• Willingness to work towards improving one of the four target health improvement behaviours and/or mental wellbeing.
**Exclusion Criteria**	• Present a serious risk of harm to the researchers or intervention practitioners;<br>• Unable to provide informed consent;<br>• Disrupted lifestyle which may make engagement in the intervention too difficult.

4 BACKGROUND AND RATIONALE

People in the Criminal Justice System (CJS) have greater physical and mental health care needs, lower psychological well-being[1] and experience significant problems in accessing health and social care services[2]. Services for those with multi-morbidities and who are under community supervision often appear fragmented[3]. A lack of trust in health services and health professionals (e.g. in primary care) causes many offenders to avoid medical help despite a high prevalence of emotional problems[4].

Unhealthy behaviours such as problematic alcohol use and smoking are much higher in the offender population than the general population[5]. For example, 60-80% of the offender population report problematic alcohol use compared to 20-30% in the general population and c. 80% of offenders smoke compared to c. 20% in the general population[6]. Both these behaviours (often co-existing) lead to several health problems, and possibly low mental well-being, through a number of plausible processes (e.g. economic, social, psychological). Likewise, substance misuse is particularly prevalent, and is also linked to mental health problems. However, services in the substance misuse field are already very well developed for offenders[7].

The Government's 2004 White Paper 'Choosing health: making healthy choices easier'[8] introduced a new workforce called Health Trainers (HTs), often drawn from the communities in which they operate. HT's main role is to provide one-to-one support to people in disadvantaged areas to facilitate health behaviour change. A handbook for HTs was developed in 2008 outlining the approach and evidence-based techniques (e.g., goal-setting, self-monitoring, creating action plans) that HTs can use to help people change behaviour[9]. The core work of HTs includes the support of behaviour changes such as healthy eating, stopping/reducing smoking, increasing physical activity, reducing alcohol and improving mental wellbeing. Their work has been positively rated but there is still a lack of robust evaluation[10].

Our rapid review of published and grey literature, and contact with local probation service leads, revealed that the scope of HTs has been extended to prison and probation settings with promising findings[11], especially when the HT has experience of the criminal justice system. While HTs have typically focused on supporting health behaviour change, there is increasing interest in their role being extended to facilitate improvements in mental wellbeing. Where enhancing wellbeing has been the main focus, individuals are more likely to attain their planned goals[11]. In parallel work, a screening and brief intervention for reducing alcohol use in individuals in the criminal justice settings[12–14] indicated no additional benefit in comparison with feedback on screening and a client information sheet[15], suggesting a more client-centred intervention with longer engagement may be needed. A recent systematic review[16] identified 95 studies working with offenders both in and out of prison (42 studies based in the community) on improving health outcomes, of which 59 led to improved mental health, substance use, infectious disease or health service utilisation outcomes, suggesting interventions can be successful. However, 91 of the studies had an unclear or high risk of bias and the review highlighted the lack of high quality rigorous research with a population which is comparatively under researched. Further rigorous research is therefore needed to evaluate the effectiveness and cost-effectiveness of a HT-led intervention aimed at improving mental wellbeing and health behaviour among people under community supervision, and to understand the change processes involved.

The current reorganisation of community supervision, as part of the 'Transforming Rehabilitation' agenda, presents opportunities to engage with those released from prison with sentences under one year (i.e. people who were previously unsupported), alongside those with community sentences, and to develop an intervention tailored to meet their needs. Providing HT support within this context could improve engagement with existing health promotion services[17], stimulate greater ownership and control over health behaviour change and involvement in activities to foster mental wellbeing[18].

There has been increasing interest in subjective wellbeing, distinct from lack of mental illness, as an important concept. The following five behaviours to increase mental capacity and wellbeing were recommended in the Foresight Report[18]: Connect with others; be physically active; take notice of things around you; keep learning; and give. Subjective wellbeing is an important outcome in its own right and has the potential to change relatively quickly. Specific health-related behaviours such as smoking, physical activity levels, alcohol intake and poor diet are well-established as risk factors for development of cardiovascular disease, diabetes and cancer and improvements in these behaviours can prevent such diseases in the longer-term. The bi-directional interactions between wellbeing and health-related behaviours are complex.

Wellbeing potentially impacts on physical health (e.g. hypertension, heart disease) and mental health (e.g. depression, self-harm, substance misuse); health behaviours (e.g. smoking, alcohol); employment and productivity; crime; and society in other ways[18]. While the role of exercise for improving well-being is clear, changing other specific health related behaviours such as smoking can also improve subjective feelings of wellbeing for some individuals. Individuals' patterns of current behaviour, motivation to change and potential benefits will be idiosyncratic and require a personal analysis. Assessing the benefit of health promotion interventions is rarely easy and wellbeing poses particular problems. One method of assessing subjective wellbeing is through the Warwick and Edinburgh Mental Wellbeing Scale (WEMWBS). The WEMWBS captures the two perspectives of mental wellbeing: (1) the subjective experience of happiness (affect) and life satisfaction (the hedonic perspective); and (2) positive psychological functioning, good relationships with others and self-realisation (the eudaimonic perspective). The latter, based on Self-Determination Theory, includes the capacity for self-development, positive relations with others, autonomy, self-acceptance and competence[19] and, therefore, the potential to positively enhance further health promoting behaviours.

The WEMWBS has been widely used at a population level to assess mental wellbeing, as well as with individuals in specific groups[20–22]. Original data we obtained from the Scottish Prisoner Service showed a mean (SD) WEMWBS score of 43.2 (12.3) (range 14 to 70), compared with a general population score of 51.6 (8.71) for England[22] and 49.9 (8.5) for Scotland[23]. Lower scores are associated with smoking, lower consumption of fruit and vegetables, high alcohol use and lower socio-economic status[24]. While these associations are likely to involve reciprocal causal effects, this does highlight the need for interventions to improve the mental wellbeing among groups with the lowest scores.

Our proposed intervention also aims to reduce specific risk factors for long-term conditions, but while these are more prevalent in probation populations, it is far from clear at this stage which of the target health behaviours, which are also long-term health risk factors, will be

selected by individuals to change. We have therefore selected WEMWBS as the likely primary outcome in the future definitive trial.

People who receive community supervision from the new National Probation Service (NPS) and CRCs services are particularly suitable for a high intensity health promotion intervention for four reasons: (1) they are often excluded from 'usual' health care and health and wellbeing-promoting interventions due to a combination of access arrangements, lifestyle factors and distrust of authority; (2) they often have low levels of mental wellbeing and poor health-related behaviours and thus the gains of the proposed intervention are potentially high; (3) while under supervision, and therefore in a period of sustained mandated contact with a service, there is an opportunity to both engage such individuals in an intervention and capture follow-up data within the context of a rigorous evaluation; (4) being subject to justice supervision can often be a time when individuals wish to improve their life circumstances, particularly towards the start of sentences.

The proposed research will develop and test the feasibility and acceptability of a client centred intervention (see 'Planned intervention' section below), for individuals receiving community supervision, to support them to change one or more health-related behaviours, enhance their wellbeing and to reduce the risk of long-term conditions. The HT role has been adapted for specific populations, including offenders[11] and smokers[25], with early signs that the support is acceptable and feasible. However, further intervention development and piloting is required to integrate a focus on promoting wellbeing and multiple health behaviour changes in offenders in the new NPS/CRCs context, and to understand the interactions between wellbeing and health behaviour changes. These uncertainties will be explored, and reduced, in a process evaluation (PE), working with the peer researchers who will have lived experience of the CJS. The pilot trial and PE will further test our assumptions, the intervention and cost-effectiveness.

The aim of the pilot trial is to provide estimates and procedures for running a future definitive trial. This pilot trial is a necessary preparatory step in ensuring maximum acceptability and feasibility for a future definitive RCT.

5 AIMS AND OBJECTIVES

The overall aims of the trial are:

1. To further develop a HT-led intervention aimed at helping people under community supervision to receive support to improve mental wellbeing and be empowered to change health behaviours.

2. To assess the acceptability and feasibility of such an intervention, alongside routine engagement with community supervision services, for the key stakeholders including CRCs, the NPS, HTs and those receiving community supervision.

3. To assess the acceptability of recruitment, assessment and randomisation procedures within a pilot pragmatic, randomised controlled trial of the intervention versus usual care (to be defined by service observation, but likely to be minimal). Determine acceptability and

feasibility of the methods in a pilot trial, including: proportion of eligible participants; recruitment rate; attrition and loss to follow-up; completion and completeness of data collection; estimates of the distribution of outcome measures; acceptability of intervention to participants; acceptability of study participation to participants.

4. To determine, from the pilot RCT, completion rates for proposed outcome measurements to assess wellbeing (WEMWBS) and behavioural measures (e.g. self-reported alcohol consumption, smoking, diet, physical activity) and quality of life (SF36 and EQ-5D-5L) at baseline and follow-up.

5. To provide data to contribute to sample size calculations for a fully powered RCT to primarily assess subjective wellbeing (WEMWBS) and to ensure that the effect size (intervention vs. usual care) chosen for powering the definitive trial is plausible.

6. To use a two-stage, mixed methods, process evaluation to refine and understand the acceptability and feasibility of the intervention, its delivery and the trial procedures. The findings will be used to refine the intervention and the logic model of the causal assumptions that underpin it.

7. To estimate the resource use and costs associated with delivery of the intervention, and to pilot methods for the cost-effectiveness framework in a full trial.

These objectives will be achieved following MRC guidance for the design and evaluation of complex interventions[26]. This involves the breakdown of the trial into an 8-month development and set-up phase (complete as of August 2016) followed by the delivery of a pilot randomised trial (September 2016-December 2017).

6 STUDY DESIGN

6.1 Summary

This protocol describes a parallel two-group randomised pilot trial with 1:1 individual participant randomisation to either the STRENGTHEN intervention plus standard care (intervention) or standard care alone (control) with a parallel process evaluation.

Following identification as being potentially suitable from NPS and CRC community supervision records, potential participants will be approached by the offender manager (OM), either when attending supervision or via the telephone (depending on current mode of contact being used in their supervision), and invited to take part in a study for those who are willing to take part in a research study and are interested in doing things that make them feel better about themselves and receiving support to improve one of the four target health improvement behaviours and/or improve their wellbeing. If interested, the potential participant will meet with the Research Assistant (RA) who will then conduct the baseline assessment. 120 participants (60 at each region, with two sites in Plymouth and one in Manchester) will be individually randomised to receive either the STRENGTHEN intervention plus standard care, or standard care alone. The STRENGTHEN intervention will be delivered over approximately 14 weeks consisting of up to 12 one-to-one sessions with a HT.

Outcome measures data will be collected at baseline (at or shortly following recruitment) and 3- and 6-months post-baseline. Six months is the proposed primary assessment point for the future definitive trial.

6.2 Setting

The study will be conducted in two regions, in the South West (Plymouth University) and in the North West (Manchester University). Participants will be recruited from CRCs in both locations, and via the NPS only in Plymouth. Conduct of the trial in each region will be led by a local Principal Investigator (PI) supported by a research team. All research staff will have an enhanced DBS check and receive training in Good Clinical Practice (GCP) and in the requirements of the study protocol.

6.3 Outcome measures

6.3.1 Acceptability and feasibility outcomes

- Proportion of eligible participants
- Recruitment rate
- Attrition and loss to follow-up
- Completion and completeness of data collection
- Estimates of the distribution of outcome measures
- Acceptability of intervention to participants
- Acceptability of study participation to participants

6.3.2 Secondary outcome measures/proposed outcomes measures for future definitive RCT

- Subjective mental wellbeing (WEMWBS)[20,21,27,28]
- Self-reported smoking (number of cigarettes smoked per day)[25]
- Fagerström Test for Cigarette Dependence (FTCD)[29]
- Alcohol use (AUDIT-C)[30]
- Diet (Dietary Instrument for Nutrition Education [DINE])[31]
- Physical activity (7-day recall of physical activity)[32]
- Substance Use (TOPS)[33]
- Confidence, importance, access to social support, action planning, and self-monitoring measures relating to health behaviours
- Health related quality of life (EQ-5D-5L, SF-36)

6.3.3 Economic outcomes:

- Key areas of intervention resource use and costs (e.g. HT time, training, supervision, travel, consumables)

- Health care, social care, and other resource use data will be collected using a participant self-report resource use questionnaire (RUQ).
- Alongside the primary clinical outcome, the primary economic outcome will be the quality-adjusted life-year (QALY) derived from the EQ-5D-5L[34,35], with the SF-36 [36] used to derive QALYs (SF6D)[37] in sensitivity analyses.

Within-trial data, collected via STRENGTHEN practitioners' records, will be used to estimate the resource use and costs associated with delivery of the intervention. Delivery of the intervention is expected to comprise HT/Practitioner time input (including contact time and non-contact time), supervision of HT/Practitioner specific to the delivery of the intervention, and costs associated with training and set-up of the intervention.

Procedures for collection of outcome data at each time-point are described in section 10 (Study Schedule).

6.4 Considerations for minimising bias

After informed consent is given and baseline data collected, participants will be allocated (1:1) to intervention or usual care trial arms via a secure, password-protected web-based system, created and managed by the United Kingdom Clinical Research Collaboration (UKCRC)-registered Peninsula Clinical Trials Unit (PenCTU) together with a statistician independent from the study team.

To minimise the chance of selection or subsequent performance bias, allocation will be concealed from the RAs at the point of allocation; the web-based system will confirm that allocation has been successfully made but will not reveal the allocated treatment arm.

The extent to which the researchers can remain blinded will be examined in the pilot trial, with researchers recording in the CRF (Case Report Form) instances where they believe they have been unblinded and what they believe the participant's allocation to be.

6.4.1 Attrition bias

Attrition bias will be minimised by having robust trial procedures to prevent data loss. The research team will make multiple and sustained attempts to follow up each participant at each time point. Procedures have been developed and tested within the Engager trial [38] for maintaining contact with participants following their release from prison and researchers will endeavour to maintain engagement with participants in between data collection points based on these procedures. The research team are also working closely with men and women with lived experience of community supervision in project Patient and Public Involvement (PPI) groups in order to develop strategies to encourage and support retention. The recent introduction of the CRCs will mean that all participants will have CJS supervision in the community and it is anticipated that this will reduce the number of participants lost to follow-up.

Recognising that this population can be difficult to follow up in the community, we will begin to contact participants well in advance of the follow-up date. It has been noted during the Engager trial pilot that contact can be lost with some participants for a period but subsequently re-established within the trial period through contact with services for whom

the research team has obtained prior consent to contact them through. If a participant misses a follow-up assessment (e.g. at 3 months), they will continue to be included in the study until all follow-up time-points have lapsed, after which they will be regarded as lost to follow-up. RAs will attempt to maintain contact via services and phone and update contact details during the study to maximise trial retention. Participants in both arms will be contacted by an RA at 3 and 6 months to collect outcome measures.

The numbers and reasons for drop-outs and losses to follow-up will be reported for each arm of the study.

6.4.2 Potential contamination between trial arms

There is unlikely to be significant contamination between the intervention and control arms of the study, although it is theoretically possible for: trainers to train other practitioners, practitioners to pass on skills and working practices to those treating control individuals, materials such as the adapted HT manual and worksheets to influence practice for control individuals, and for participants to influence each other. The extent to which possible contamination may occur through participants having existing relationships with each other (e.g. cohabiting) will be captured in the pilot trial and, if noted, its possible influence examined within the process evaluation.

The risk of contamination is considered low, primarily because there is no alternative funded pathway for delivery of the substantive components of the intervention for those in the control arm. STRENGTHEN practitioners will form a separate team within and alongside the NPS and CRCs and, while other practitioners will be informed about the intervention, they will not be trained in the details.

In order to further mitigate risk of contamination we will give clear instructions to the intervention practitioners not to provide manuals or supplementary intervention materials to any participants not assigned in the Intervention group. HTs will also be instructed not to supply materials or recommend techniques to colleagues who may be providing usual care.

7 STUDY PARTICIPANTS

7.1 Participants

7.1.1 Inclusion criteria

Participants must satisfy the following criteria to be enrolled in the study:

- Males and females aged 18 years or older;
- Receiving community supervision;
- For prison releases, have been in the community for at least 2 months;
- A minimum of 7 months left to serve of community sentence;
- Willing and able to receive support to improve in one or more of the four target health behaviours and/or improve wellbeing;
- Willing and able to take part in a pilot randomised controlled trial with follow-up assessments at 3 and 6 months;
- Residing within the geographical areas of the study.

7.1.2 Exclusion Criteria

Participants who meet any of the following criteria will be excluded from study participation:

- Those who present a serious risk of harm to the researchers or intervention practitioners;
- Those unable to provide informed consent;
- Those with disrupted lives who may find it difficult from the outset to engage in the intervention.

8 STRATEGIES FOR PATIENT IDENTIFICATION

8.1 Database search

Potential participants will be identified in partnership with the CRCs and NPS. We will work with these services and their new and developing record-keeping systems to identify potential participants who have at least seven months of community supervision left to serve and, if recently released from prison, have been in the community for at least two months. We will also work with these services to identify and exclude potential participants who present a serious risk of harm to the researchers or intervention practitioners. Community organisations including drug and alcohol rehabilitation centres, homeless hostels and day centres, will also support initial identification of potential participants.

Decisions whether or not to include someone, based on their level of risk, will be taken by the research team at each site in conjunction with local services if needed.

8.2 Initial approach and provision of study information

A single point of access (SPOA) administrator has been identified for both CRC and NPS. The SPOA administrator will identify potential participants from the nDelius record system for both services. OMs of identified individuals will be consulted for screening for inclusion/exclusion criteria and assessment of risk. On receipt of clearance to approach potential participants, OMs will ask clients if they would agree to speak to the site researcher either at their next scheduled appointment or via the telephone (depending on the current mode of contact between the OM and potential participant within their community supervision). On receiving verbal agreement to approach, the OM will facilitate the researcher to make the initial approach either in person, following the individual's routine appointment at CRC/NPS or via the telephone. All potential participants will be offered to meet the researcher for the initial appointment in a meeting space at CRC/NPS offices. Identification of participants through community organisations will involve initial staff approaching potential participants to invite them to talk to a researcher about the study. On receiving verbal agreement to approach, the researcher will make a time and date for a meeting. The researcher will explain the study and provide the opportunity to ask questions. If the individual expresses an interest in taking part in the study, the researcher will progress with the consent process.

9 STUDY SCHEDULE

This section describes the conduct of the study in chronological order, detailing procedures for data collection at each of the time points. A tabulated summary of the study schedule is given in Table 1 below. This section does not describe collection of additional process evaluation data. The process evaluation data collection procedures are described in section 14.

9.1 Baseline visit

9.1.1 Consent Process

Following the initial approach, if a potential participant expresses an interest in taking part in the study, a meeting will be arranged between the researcher and the potential participant where the researcher will explain the project in more detail. This meeting may take place immediately after the initial approach, but the potential participant can take longer (a minimum of 24 hours) to consider if they want to take part if necessary.

The researcher will give the participant a copy of the Participant Information Sheet (PIS) at the meeting. The researcher will read and explain the information in the PIS, showing sensitivity to the high levels of literacy difficulties in this population. The researcher will explain what participation in the study involves and how much time will be involved. The researcher will ensure that the potential participant fully understands what randomisation means and that they have an equal chance of being randomised to either the STRENGTHEN Intervention Group or the Control Group. They will also explain that participation is voluntary, that they can withdraw at any time and at any point and that their decision to participate, or not, will have no adverse effect on the care that they receive or their other legal rights. The researcher will also discuss the arrangements to ensure confidentiality (and limits of this) and data protection. Throughout this process, the potential participant will be given an opportunity to ask questions. Potential participants will be made aware of circumstances in which confidentiality would be broken.

Having had the opportunity to discuss their involvement in the study and ask questions about it, potential participants will be asked to sign the consent form if they are:

- Willing and able to receive support to improve one of the four target health behaviours and/or improve mental wellbeing;
- Willing and able to take part in a pilot randomised controlled trial with follow-up assessments at 3 and 6 months;

If a potential participant is unwilling or unable to proceed they will be thanked for their time and contribution and their involvement will end. If a potential participant is both willing and able to proceed to the trial, the consent form will be explained to them before they sign it and the researcher will sign the form after it has been completed by the participant. A copy of the signed consent form will be given to the participant and a copy will be retained by the researcher.

Participants initially identified through CRC/NPS: The researcher will then continue with the baseline data collection during this same visit/meeting, checking that the participant is happy to proceed.

Participants initially identified through community organisations: The consent form for participants who are identified through community organisations requests consent for the researcher to make contact with the participant's OM in order to check that they meet the criteria for participation in the study. Following positive assessment by the OM, the researcher will contact the participant to make a time to conduct the baseline data collection. If the OM assesses the participants as not meeting the criteria for inclusion in the study, the researcher will make a time to explain why the participant in unable to take part in the study.

Table 1: Tabulated summary of study schedule

	**BASELINE ASSESSMENT**				
	**Screening**	**Baseline Data**	**Allocation**		
**TIMEPOINT**	t_1	t_1		***+3 mth*** T_2	***+6 mth*** T_3
**ENROLMENT:**					
**Eligibility screen**	X				
**Informed consent**	X				
**Allocation**[1]			X		
**INTERVENTIONS:**					
Intervention Group: ***Strengthen Intervention***					
Intervention Group: ***Usual care***					
Control Group: ***Usual care***					
**ASSESSMENTS:**					
Demographics		X			
WEMWBS		X		X	X
AUDIT-C		X		X	X
DINE		X		X	X
7 Day PA recall		X		X	X
Sefl Reported Smoking		X		X	X
FTCD		X		X	X
Importance, confidence, social support, action planning, self monitoring		X		X	X
Treatment Outcomes Profile (TOP)		X		X	X
EQ-5D-5L Questionnaire		X		X	X
SF36		X		X	X
Resource use questionnaire		X		X	X
**SAFETY MONITORING:**					
Adverse event reporting	◆———	———	———	———	———◆

[1] Allocation will be performed using a web-based system provided by the CTU, usually within 2 days of completing the baseline and written consent being obtained.

9.1.2 Baseline data collection (t_1)

The researcher will normally continue with the baseline data collection following screening; additional sessions can be arranged to meet the needs of individual participants if necessary. If the baseline data collection occurs more than two weeks after initial screening, a rescreening will take place prior to baseline data collection.

The researcher will continue to deliver the baseline data collection assessment using the narrative conversational format developed in our previous studies. The questions from the WEMWBS (the primary outcome) will be read out to participants in a precise and consistent manner. Questions from other measures are incorporated into a specially constructed flexible script which avoids duplication of subject matter in order to reduce disengagement or irritability:

Data will be recorded in the Baseline CRF.

In addition to the baseline data collection, the researcher will complete a contact sheet for each participant. This will include contact numbers and addresses provided by the participant, as well as a list of services they are likely to be in contact with. This sheet will be completed in collaboration with the participant and the participant will sign the form to confirm they give the research team permission to contact them via the relevant services.

9.1.3 Randomisation process

Allocation to intervention or control group will use minimisation, with a random element, to ensure balance between treatment arms with respect to age, gender and recruitment site. Recruitment site will be determined by a combination of geographic region and the service type: 1) Manchester CRC; 2) Plymouth CRC; 3) Plymouth NPS. Recruiting from Manchester NPS is not possible in this study and so it is not possible to minimise on both geographic region and service type. Full details of the allocation process will be documented separately. Allocation will be achieved by means of a web-based system created by PenCTU.

Once the participant has completed the screening interview and baseline data collection assessment, the researcher/administrator will subsequently access the randomisation website using a unique username and password. The website will require entry of the study site, participant initials, participant age and gender, before returning the participant's unique randomisation number and allocation (STRENGTHEN Intervention or Control) to the trial administrator via email. The website will confirm that the allocation process has been successful but will not display the participant's allocated group at the point of entry, to maintain blinding of the RAs.

9.1.3 Communicating allocated group to participants

To maintain blinding of the RAs who will be collecting outcome data, research administrators will telephone participants allocated to the control arm to inform them of their allocated Group. HTs will contact those allocated to the intervention arm. The research administrator or HT

will go through the results of the randomisation process with the participant, ensuring that they understand which group they are in.

The intervention practitioners will be sent, via encrypted and password protected email, a pseudo-anonymised two-page ‘referral form’ by the researcher team for each participant randomised to the STRENGTHEN Intervention. The referral form will contain the participant unique ID number, along with contact and demographic details.

9.2 3-month outcome measure collection (t_2)

The blinded researcher will contact the participant. This data collection point can be completed via a phone call, but will preferably be done face-to-face to support continued engagement. If collecting data is highly problematic, attempts will be made to collect a minimum data set containing at least the primary outcome.

The questions from the following measure will be read out to participants:

- WEMWBS;
- AUDIT;
- DINE;
- Self-reported smoking;
- FTCD;
- TOPS;
- 7-day PA recall;
- Confidence, importance, social support, action planning, and self-monitoring;
- EQ-5D-5L;
- SF36;
- Resource Use Questionnaire.

The researcher will discuss the 6-month follow-up in detail and agree the best way to contact the participant for that appointment, depending on a range of scenarios, and changes to modes of follow-up, including any new mobile telephone numbers.

9.3 6-month outcome measure collection (t_3)

Researchers will arrange to meet the participant at a convenient location in the community. Where possible, assessments will be conducted in the premises of services that the participant is engaging with, in order to minimise risk to the researcher. Where this is not possible, researchers will arrange to conduct the assessment in a suitable location in the community and adhere to the Lone Working policy. Buddies may be used as an additional safeguard.

The researcher will remind the participant of the information sheet and consent process, drawing attention to data confidentiality and instances of disclosure where the researcher would need to breach confidentiality.

If collecting data is problematic, attempts will be made to collect a minimum data set containing at least the primary outcome; this may be done by telephone.

10 MINIMISING ATTRITION

It is recognised that many of the participants will have chaotic lifestyles and it will be a challenge to maintain their engagement with both the Intervention and the research elements of the study. Pilot work within the Engager trial has demonstrated that this population are not always contactable, but often become re-contactable at a later stage.

The following steps will be taken to attempt to minimise attrition:

- Work closely with NPS and CRCs and their developing data systems;
- Send SMS ahead of 3- and 6-month follow-up;
- Ask participant to send change of contact details as appropriate.

If a participant cannot be contacted and misses the 3-month follow-up assessment, they will not be withdrawn from the study and researchers will continue to try to contact them until the end of the 6-month follow-up window.

10.1.1 Return to prison

Return to prison is not a reason for automatic withdrawal from the study. Any participant who returns to prison will continue to be included in the research and, where possible, the researchers will attempt to conduct follow-up assessments in the prison where they are detained. Relevant permissions and associated amendments to approvals will be requested where required.

The location of the follow-up assessment (prison or community) will be documented.

11 INTERVENTION

11.1 Description

Through original research and literature reviews, we have developed an extensive understanding of what are likely to be the effective components of an intervention targeted at health behaviours and improvement of health and wellbeing in this population. A clear starting point logic model of intervention components and aims underpins the intervention, based on the HT role in a previous trial of smoking cessation in disadvantaged groups and the development of a collaborative care model for prison leavers with multiple health problems. The intervention aims to enhance people's mental wellbeing, improve their health-related behaviours and, eventually, reduce the risk of long-term conditions (e.g. depression, diabetes, CVD, cancers). This will be achieved through participants doing things that make them feel better about themselves and improving their health, supported by the '5 Ways to Wellbeing' (5WWB)[18] and/or working towards one of the four target health behaviour changes (smoking reduction, alcohol consumption reduction, increasing physical activity or improving healthy eating), supported by the HT. The relationship between mental wellbeing and the target health behaviours is interactive and bi-directional. Our key uncertainties concern the main pathways of involvement and the influence of social environment. Some participants may feel satisfied with their levels of wellbeing and focus on their chosen target behaviour, some may need to improve their mental wellbeing before working towards their target behaviour; others may work towards both. We are currently working alongside Peer

Researchers (individuals with lived experience) to reduce these uncertainties and will test them further within the pilot trial process evaluation.

A training package is being delivered to the HTs on the project, through which they will demonstrate the core competencies of a HT as outlined in the HT Handbook[9], and receive training in 5WWB, both for improving their own awareness and for knowledge in passing on the benefits, reflecting the principle that 'if you don't have it, how can you give it?'[39]. During the manualisation phase, the HT handbook will be adapted to incorporate the principles of 5WWB and be tailored for working with the target population.

The key components of the planned intervention are:

1. A HT will be available for up to 12 client-centred one-to-one sessions (over 14 weeks), in face-to-face or telephone format; we expect an average of 4-6 sessions (with greatest results being achieved up to 6 sessions with diminishing returns beyond that[40]). The face-to-face intervention sessions will take place in a variety of settings, including probation services and other local community locations. Initial engagement and proactive follow-up is based on our previous offender research.

2. An initial invitation to engage with the HT will describe an 'open and flexible' opportunity to receive support for one of the target health behaviours and/or improving overall health and mental wellbeing through other activities including connecting, keeping learning, being active, taking notice and giving.

3. HTs will be trained to help participants to understand the inter-relationship between health behaviours such as smoking, alcohol use, diet, physical activity and their relationship to mental wellbeing and other positive and negative behaviours, including substance use. Each participant will develop a personal plan based on individual behaviour change goals and motivation to improve mental wellbeing. Some offenders will have positive perceived mental wellbeing but engage in negative behaviours, others will be as concerned about emotional distress. The intervention intends to be flexible enough to support both these extremes.

4. The support is described as 'open' to reflect the planned underpinning and overlapping influence of Self-Determination Theory and the client-centred principles of Motivational Interviewing[41]. HTs will avoid giving 'advice' unless requested but empower clients to confirm the desire for change, and develop self-regulatory skills such as self-monitoring, setting action plans and reviewing progress. The intervention will be individually tailored and led by the participants' needs.

5. The HT will, informed by the 5WWB, help clients to build positive behaviours (e.g. initiating and maintaining activities (physical, creative etc.) and finding opportunities for gaining core human needs (i.e. sense of competence, autonomy and relatedness), as well as learn and notice, to enhance psychological wellbeing.

6. Any reductions in alcohol consumption (as units per week, alcohol-free days, or avoidance of trigger events[42]), smoking (using different strategies[25,43]), and increases in physical activity and healthy eating will be supported, with the aim to build confidence to meet guidelines for safe alcohol consumption, to quit/reduce smoking, engage in daily/weekly physical activity, and healthy eating.

7. Participants will be actively supported to gain help from friends and family, link with other community resources (parks, leisure centres) and services (e.g. Stop Smoking Services, Drug and Alcohol Treatment Service) as a part of achieving their personal plan, exploring options for continued support after the intervention as appropriate. We have found signposting alone to be insufficient with this population[25].

11.2 Delivery

The HTs will meet with participants at a location acceptable to both parties, which is likely to be within offices of the NPS or CRCs initially. Other intervention sessions can take place within local services and support centres and over the telephone. Intensity and frequency of support will be dictated by the individual's needs and preference, with up to 12 sessions being offered over up to 14 weeks.

Overall components

The components of the implementation platform include:

- A manual describing actions for practitioners;
- A training programme for practitioners;
- A programme of supervision put in place for practitioners;
- A set of organisational agreements;
- Other equipment and tools.

Manual

A comprehensive manual will be produced in Phase 1 to guide practitioners in following components of the intervention.

Training

The practitioners taking on the role of the STRENGTHEN HTs will have no previous formal training as therapists and will likely come from a variety of backgrounds. They will have some experience of some combination of coaching, problem-solving, supporting others to change or motivational approaches.

Practitioners will be trained in the logic and rationale of the model and behaviour change techniques. They will receive additional training in motivational interviewing approaches to support the delivery of the intended intervention objectives. The training will be based on the core HT competencies, and adapted to include the incorporation of the 5WWB.

Supervision

Supervision will be conducted by a member of the research team (TT) who will provide weekly supervision on an individual basis with each HT. Monthly meetings will take place with all HTs together (virtually across geographical locations) in order to provide a formal opportunity for shared experiences and challenges. The supervisor will also listen to and analyse recordings of intervention delivery sessions on a bi-weekly basis which will be scored against a delivery fidelity checklist to help identify deviations from the protocol as well as provide formative feedback to the HTs and identify any ongoing need for re-training to ensure strong delivery fidelity.

Organisational agreements

A set of organisational agreements will be put in place in order to ensure individual practitioners receive a supportive context and are able to practice safely. These include honorary contracts for individuals to be able to work as part of other organisations, information sharing/confidentiality agreements, and less formal agreements to house and support practitioners with desk space, computers, etc.

Other components

A range of other physical objects important to the delivery of the intervention have been/will be developed. These include worksheets for practitioners to work with individuals (also forming the appendix to the manual), mobile phones, and office and desk space.

11.3 Withdrawal from intervention

Lack of response to contact will not be taken as an indication for withdrawal. Practitioners will continue to attempt to make contact. Practitioners will review the number and types of contact attempts on a case-by-case basis with their supervisor, to avoid harassment. Withdrawal from the intervention can however be initiated at any time by the participant. Those withdrawing from the intervention will still be included in follow-up unless they also ask to be withdrawn from the research; their right to do so will be made clear to them.

11.3.1 Return to prison

Should a participant be incarcerated whilst receiving the intervention, the support will still be available for up to 14 weeks post-baseline, should they be released and able to engage in the intervention again within this timeframe. If they are incarcerated and released beyond this time frame, they will not be eligible to be recruited again into the study should their release fall within the study recruitment period. Each case of a participant being unable to continue with the intervention will be reviewed on a case-by-case basis.

12 CONTROL GROUP

Individuals in the Control Group will receive treatment as usual, which will include support from the CJS and any other third sector organisations in the standard way. For each site we will identify what support participants would normally receive, whilst working with the NPS and CRCs, and this will be documented and maintained. Participants in both arms of the study will have access to all local services as usual.

13 PILOT TRIAL PROCESS EVALUATION

Aims

1. To assess whether the intervention is being delivered as per manual and training;
2. To ascertain components of intervention which are critical to delivery;
3. To explore reasons for divergence from delivery of intervention as manualised;
4. To understand when context is moderating delivery;
5. To understand the experience and motivation of participants in Control arm of pilot in order to maximise retention in a full trial;
6. To explore reasons for declining to participate in the trial;
7. To explore reasons for disengaging in intervention before an agreed end;
8. To understand, from a participant perspective, the benefits and disadvantages of taking part in the intervention.

Data collection:

Data collection will be conducted using a variety of sources in order to meet the aims of the Process Evaluation (PE) above:

1. **Semi-structured 1:1 interviews:**

1:1 interviews will be conducted with:

- HTs (n=6 – part-time) across both geographic regions;
- Offender Managers/probation worker (n=6) across both geographic regions
- Participants who disengaged before an agreed end (up to 6);
- Participants randomised to Intervention arm of pilot (n=6);
- Participants randomised to Control arm of pilot (n=6).

All Interviews will be digitally audio-recorded and transcribed verbatim.

2. **Discussions with decliners:**

The Research Assistant will ask up to 4 potential participants who decline participation following screening as to their reasons for not continuing with their participation. The RA will be sensitive to the right to withdraw from the study without providing a reason and will not question the potential participant further should they decline to divulge their reason for discontinuation. These discussions will not be recorded and rather notes will be taken to inform the PE.

3. **Digital audio recordings of HT sessions (n=20)**

HTs will be asked to record sessions with participants by the research team. Choice of sessions to record will be a collaborative decision between the HT and the research team based on appropriateness (to be assessed by the HT) and data required (to be assessed by the research team and guided by their knowledge of each case through HT session report forms. All participants will have been asked for their consent for sessions to be recorded at the start of the intervention. However, HTs will be requested to seek verbal consent to record each session prior to recording.

4. **HT session report forms**

HTs will be asked to keep a log and record of each session, including information on: date, location, duration, type (face to face or by telephone), subsidies taken up by participant, primary goals of participant, goals met (if applicable), and any particular difficulties encountered for discussion in supervision.

Analysis:

- Intervention fidelity will be assessed through the scoring of audio recordings of HT sessions against a developed list of key intervention processes (drawn from the logic model). These will be scored on two domains: practitioner adherence to the protocol, and for competence of delivery;
- Quantitative data will be summarised descriptively, with confidence intervals as appropriate. Any factors which are identified as possibly contributing to participants' intervention engagement, and trial recruitment and retention will be explored in more detail in the qualitative data; Data from these sources will be synthesised into a Framework Analysis grid supported by Nvivo 10 software[44]. Framework analysis will allow the feasibility and acceptability of the intervention, the intervention delivery and the research data collection to be assessed. Any procedures which need to be adapted will be identified and, potentially, improvements and solutions will be suggested.

Contribution:

The PE will contribute to the research through:

- Revision of logic model, intervention, intervention delivery and research data collection for full trial;
- Identification of which areas of the intervention are not being delivered as intended to help plan for future training and development in a definitive trial;
- Any generalisable learning about the feasibility and acceptability of trial procedures with this population. This will be shared via a journal publication.
- The decision as to whether to progress to a full trial or not;
- The design of the PE for full trial. If minimal changes are made to the intervention, the intervention delivery and the trial procedures this data could be considered to be part of an internal pilot and the data could be added to similar data from an external pilot to form the data for a full trial PE.

14 SAFETY REPORTING

14.1 Definitions

Adverse Event (AE)

Any untoward medical occurrence, unintended disease or injury or any untoward clinical signs (including an abnormal laboratory finding) in participants **whether or not related to any research procedures or to the intervention.**

Seriousness

Any adverse event will be regarded as serious if it:

i. results in death;
ii. is life threatening;
iii. requires hospitalisation or prolongation of existing hospitalisation;
iv. results in persistent or significant disability or incapacity;
v. consists of a congenital anomaly or birth defect; or
vi. is considered by the investigator to be an important medical event

An adverse event meeting any one of these criteria will be a **Serious Adverse Event (SAE)**.

Relationship

The expression 'reasonable causal relationship' means to convey, in general, that there is evidence or argument to suggest a causal relationship. The research team will assess the causal relationship between reported events and trial participation according to the standardised guidance given below:

Relationship	***Description***
Unrelated	*There is no evidence of any causal relationship.*
Unlikely	*There is little evidence to suggest there is a causal relationship (e.g. The event did not occur within a reasonable time after administration of the trial treatment/procedure). There is another reasonable explanation for the event (e.g. The*

	participant's clinical condition, other concomitant treatment).
Possible	*There is some evidence to suggest a causal relationship (e.g. Because the event occurs within a reasonable time after administration of the trial treatment/procedure). However, the influence of other factors may have contributed to the event (e.g. The participant's clinical condition, other concomitant treatments).*
Probable	*There is evidence to suggest a causal relationship and the influence of other factors is unlikely.*
Definitely	*There is clear evidence to suggest a causal relationship and other possible contributing factors can be ruled out.*

14.2 Reportable events

We do not expect participants to experience any serious adverse events (SAEs) as a direct result of taking part in this trial. Any non-serious adverse events (regardless of relatedness) will not be reported in this study. Reportable events will therefore be restricted to only those meeting the criteria for Serious Adverse Events as defined above.

The CI will review information collected by either the HTs or the researchers which they think may be beneficial for the other parties to know, for example, if a participant has had an acrimonious break-up with their previous partner whose information is listed in their contact details. This information will be shared between the researchers and HTs, as appropriate, based on the CI's judgement and discretion.

14.2.1 Reporting Serious Adverse Events

RAs will question participants about adverse events at each of the follow-up time points. Any serious adverse events will be reported by the RA to the CTU within 24 hours of becoming aware of the event, using a trial-specific SAE report form. The report form will include a description of the event and the RA's assessment of causality i.e. whether there is a reasonable causal relationship between the event and the intervention*. The CTU will maintain a register of all reported SAEs and will routinely inform the CI by email of all reported SAEs.

For events assessed as having no reasonable causal relationship, CTU will obtain a second assessment of causality from the Chief Investigator or independent person if warranted. The Chief Investigator or nominated deputy will assess the expectedness of any events which are deemed to have a causal relationship (either after initial or second assessment).

Safety monitoring will be facilitated by regular review of cumulative SAE reports by investigators at the Study Group meetings and then the Trial Steering Committee SAEs that are related and unexpected are to be reported to National Research Ethics Service (NRES) within 15 days.

We will develop working protocols with the NPS and the CRCs to assess and address potential and any actual harm. We recognise that risk is dynamic and can escalate or decrease. We will work with the NPS and CRCs to develop good practice and ongoing risk levels, to self and others, of participants.

Detailed guidance for the reporting and processing of SAEs will be provided to study personnel by the STRENGTHEN research team in a separate work instruction.

*If incomplete information is available at the time of reporting, all appropriate information relating to the serious adverse event should be forwarded to the CTU as soon as possible.

DATA MANAGEMENT

14.3 Study Numbering

Each participant will be allocated a unique study number and will be identified in all study-related documentation by their study number and initials.

14.4 Data Collection

Data will be recorded on study specific data collection forms (CRFs), usually by the research team at each site. All persons authorised to collect and record trial data at each site will be listed on the study site delegation logs, signed by the relevant PI. Source data will include all data recorded straight into the CRF.

For the process evaluation, audio files and transcriptions of the data will be collected by the Process Evaluation Team, comprising STRENGTHEN team co-applicants, staff and collaborators.

14.5 Data entry

Completed CRFs will be checked and signed at the research sites by a member of the research team before being sent to the PenCTU. Original CRF pages will be posted to the PenCTU at agreed timepoints for double-data entry on to a password-protected database, with copies retained at the relevant study site.

All forms and data will be tracked using a web-based trial management system. Double-entered data will be compared for discrepancies using a stored procedure. Discrepant data will be verified using the original paper data sheets.

14.6 Data Confidentiality

Participant names and addresses will be collected for the purpose of managing questionnaires, intervention delivery and process evaluation interviews. Investigators will ensure that the participants’ anonymity is maintained on all other documents. Within the PenCTU, anonymised and identifiable study data will be stored separately, to prevent the identification of participants from research records, in locked filing cabinets within a locked office. Electronic records will be stored by the CTU in a SQL Server database, housed on a restricted access, secure server maintained by Plymouth University. Data in the database will be backed up daily by the Plymouth University Plymouth web team and will be accessible for up to 6 months. The website will be encrypted using SSL. Data will be collected and stored in accordance with the Data Protection Act 1998. Direct access to the trial data will be restricted to members of the research team and the CTU, with access granted to the Sponsor on request. Access to the database will be overseen by the CTU data manager and trial manager. Copies of original study data retained at study sites will be securely stored for the duration of the study prior to archiving. Audio recordings will be stored on a restricted access, secure servers at Plymouth University.

14.7 Archiving

Following completion of trial data analysis, the Sponsor will be responsible for archiving the study data and essential documentation in a secure location for a period of 5 years after the end of the trial. No trial-related records should be destroyed unless or until the Sponsor gives authorisation to do so.

15 DATA ANALYSIS CONSIDERATIONS

15.1 Sample Size

In phase 1 (developing the intervention and training; months 1-8 months), as part of the FPE we will interview CJS staff (n=6), HTs in other services (n=6), and intervention Health Trainers after they have participated in our training (n=6). We will also work collaboratively with up to 15 people with lived experience of being subject to the CJS, at the Plymouth region, to receive input into the content of the intervention and HT training manual (e.g. feasibility and acceptability of the intervention, recruitment processes for the trial, training).

In phase 2 (pilot RCT) a formal sample size calculation based on considerations of power is not appropriate; this pilot study is not powered to detect between-group clinically meaningful differences in a primary outcome. The aim is to provide robust estimates of the likely rates of recruitment and follow-up, as well as provide estimates of the variability of the proposed primary and secondary outcomes to inform sample size calculations for the planned definitive trial.

When data from a pilot study are required to estimate the standard deviation of a continuous outcome, to maximise efficiency in terms of the total sample size across pilot and main trials, the recommendation is that a two-group pilot study should have follow-up data from at least 70 participants (i.e. 35 per group)[45]. When considering binary outcomes a total of at least 120 participants is recommended[45]. For the pilot RCT (phase 2), we believe that over 3 months, and across the two sites, we will be able to approach around 330 potential participants. We aim to recruit at least 120 participants across the two geographic regions (60 per region). Local services have suggested that over a 3-month window, there may be 20-30 ex-offenders entering each of the two local community supervision systems per week; we estimate that around 10% will decline to participate in a baseline assessment[7,46] and a further 20% will be found to be ineligible following the baseline assessment. Based on recruitment rates from other probation trials[11] we estimate that around 50% of eligible subjects will consent to participate. As most participants will remain engaged with the probation service for the length of the trial, it is anticipated that retention will be reasonably high. Assuming a 6-month follow-up rate of 75%, this should provide follow-up outcome data on a minimum of 45 participants in each of the allocated groups across both sites. A follow-up rate of 60% should still provide sufficient data for planning the future trial.

15.2 Statistical analysis

An initial analysis at month 18 for the progression report will focus on 1) recruitment and retention and 2) adherence to the intervention:

1) A ConSORT (Consolidated Standards of Reporting Trials) diagram will provide detailed description of numbers approached, meeting eligibility, having baseline data collected, being randomised, and having follow-up data collected;
2) A descriptive analysis will report on the proportions of those randomised to the intervention and who; attended 2 or more sessions, completed all sessions and set behaviour change goals in personal plans.

Data from screening, recruitment and follow-up logs will be used to generate realistic estimates of eligibility, recruitment, consent and follow-up rates in the study population (objective 3), to assess the feasibility outcomes of the study. We will also estimate completion rates for each of the proposed outcome measures at each time-point (objective 4). All such estimates will be accompanied by appropriate confidence intervals, to allow conservative assumptions to be made in the planning of the definitive trial. Individuals lost to follow-up will be compared to those who complete the pilot study to identify any potential bias.

It is inappropriate to use pilot study data to formally test treatment effects, therefore the statistical analyses will be of a descriptive nature[47,48]. We will follow the anticipated ConSORT extension for reporting of pilot and feasibility studies[48,49] and take note of the ConSORT extension for reporting of patient-reported outcomes[50]. Descriptive statistics of the proposed primary and secondary outcomes will be produced, as appropriate for each measure for each group. Interval estimates of the potential intervention effects, relative to usual care, will be produced in the form of a 95% confidence interval, to ensure that the effect size subsequently chosen for powering the definitive trial is plausible, but no formal hypothesis testing will be undertaken of the pilot data[47] (objective 5). Analyses will be on an intention-to-treat basis.

16 ECONOMIC EVALUATION

The pilot study will be used to estimate the resource use and costs associated with the delivery of the intervention, and to develop a framework for estimating the cost effectiveness of the STRENGTHEN intervention plus usual care, versus usual care alone, in a future economic evaluation alongside a fully powered RCT. We will develop and test economic evaluation methods for the collection of resource use data, and for estimating related costs, and also on the collection of outcome data appropriate for economic evaluation. In a future full economic evaluation, it is anticipated that the primary perspective for analyses will be that of the NHS and Social Care Services (i.e. Third Party Payer), with a broader participant and societal perspective explored in sensitivity analyses, and this will guide the methodological framework in the pilot study research on economic analysis.

The key areas of resource use and costs associated with the delivery of the intervention will be identified (e.g. HT time, training, supervision, travel, consumables), and methods tested for the collection of data. This will be via within-trial participant level records of HT/Practitioner input (including contact time, and non-contact time). Data on participant health service use, social care service use, and other broader aspects of resource use will be collected using self-report (interviewer administered) questionnaires at baseline, 3-month and 6-month follow-ups. This resource use questionnaire (RUQ) will be developed for this participant population, using the approach described for the Client Service Receipt Inventory (CSRI[51]), and based on our experience of collecting resource use data in a wide range if prior studies.

In a future full economic evaluation cost effectiveness analysis will present the incremental cost per unit of change on the primary outcome measure (expected to be the WEMWBS). However, the primary economic endpoint, with most policy relevance, will be the incremental cost per QALY gained. QALYs will be estimated using participants data collected using the EQ-5D-5L[34],and the recommended value set for England[35]. Given uncertainty associated with estimating QALYs the SF-36, from which the SF-6D can be derived[37], will also be used to estimate QALYs in sensitivity analysis. EQ-5D-5L and SF-36 data are collected at baseline, 3-month and 6-month follow-ups, and the pilot study will assess the feasibility of use and completion rates of these measures.

A future economic evaluation is expected to include extrapolation from the trial outcomes to extend a trial-based cost-effectiveness analysis over the longer term, for example using one- and two-year time horizons. Such mathematical modelling would involve evidence synthesis, use of assumptions, and would introduce further uncertainty, and the pilot study research will be used to consider these issues and to develop the broader framework for cost effectiveness analyses, alongside a future trial. Pilot study research, as well as the estimation of intervention costs, will include exploratory and descriptive analyses on the potential incremental costs and outcomes associated with a comparison of the STRENGTHEN intervention, plus usual care, versus usual care alone. Such exploratory research will include use of extensive sensitivity and scenario analyses, with transparent

reporting to allow interpretation in a policy setting, with findings set out in a policy relevant context, for example using a cost-consequences analysis approach, which presents costs and outcomes in a disaggregated, tabular format[52,53].

17 DATA MONITORING AND QUALITY ASSURANCE

The PI (or authorised delegate) will check completed CRFs for missing data or obvious errors before the forms are sent to the PenCTU. Data will be monitored centrally for quality and completeness by the PenCTU and every effort will be made to recover data from incomplete forms where possible. The PenCTU data manager will oversee data tracking and data entry and initiate processes to resolve data queries where necessary. The trial manager (LC) will devise a monitoring plan specific to the study which will include both central monitoring strategies and study site visits as appropriate. Procedures specifically conducted by the PenCTU team (e.g. randomisation, data entry, data management) will be conducted in compliance with PenCTU SOPs.

Participating sites will be required to permit the trial manager or deputy, or representative of the sponsor, to undertake study-related monitoring to ensure compliance with the approved study protocol and applicable Standard Operating Procedures (SOPs), providing direct access to source data and documents as requested.

All study procedures will be conducted in compliance with the protocol and according to the principles of the International Conference on Harmonisation Good Clinical Practice (ICH GCP).

18 STUDY ORGANISATIONAL STRUCTURE

Responsibility for the trial is assumed by the CI (Prof. Adrian Taylor) who will ensure its timely completion. The Principal Investigators in each region will be responsible for managing all aspects of the study at their site(s).

Randomisation, study database and data management services will be provided by the UKCRC-registered PenCTU.

18.1 Project Management Group (PMG)

A PMG including the CI, trial manager, trial statistician, health economist, process evaluation team, PIs, and other relevant personnel (e.g. other clinical colleagues, CTU data manager and patient representatives) will meet regularly throughout the duration of the trial to monitor progress, resolve day-to-day problems, oversee development of documentation and forms, monitor participant recruitment and follow-up, review the budget, discuss analysis, results, draft reports and dissemination. The PMG will meet at least every quarter. The CI, PIs and trial management team will also have teleconference meetings on a monthly basis.

18.2 Trial Steering Committee (TSC) responsibility

The TSC for the study will oversee the conduct and safety of the trial. A charter describing the role and function of the committee specific to this study will be developed and agreed prior to, or soon after, study commencement. The Committee includes an independent chair,

independent members, Patient and Public Involvement (PPI) representatives and the CI Representatives from both the Sponsor and funding organisations will be invited to study-related elements of the TSC meetings as observers. The TSC will meet to approve the protocol ahead of an Ethics submission, after 6 months and then annually. Minutes of the TSC meetings will be sent to the Sponsor.

18.3 Data Monitoring Committee (DMC)

The necessity for a Data Monitoring Committee will be decided by the TSC at the inaugural meeting.

19 DIRECT ACCESS TO SOURCE DATA AND DOCUMENTS

The PI and the Sponsor will permit trial-related monitoring, audits, regulatory inspections and REC review by providing appropriate bodies (e.g. PenCTU, REC etc.) direct access to source data.

20 RESEARCH GOVERNANCE

20.1 Sponsor

The research is sponsored by Plymouth University, represented by Ms Pam Baxter, Research Governance Officer.

20.2 Ethics and NHS approvals

The study will be conducted in accordance with the Research Governance Framework for Health and Social Care, Second edition (2005)[54] and approved by a recognised NHS REC, and the Trust R&D Departments for each region. The study will be adopted by the National Institute of Clinical Research (NIHR) Clinical Research Network (CRN).

The trial will be conducted in accordance with the ethical principles that have their origin in the Declaration of Helsinki, and that are consistent with GCP. Any amendments to the protocol will be submitted for REC approval as appropriate.

On request, the Chief/Principal Investigators will make available relevant trial-related documents for monitoring and audit by the Sponsor, and the relevant Research Ethics Committee.

Annual progress reports will also be submitted to the REC using the recognised NRES template. An end-of-trial declaration will be provided to the REC within 90 days of trial conclusion or within 15 days of trial termination in the event the trial is prematurely terminated.

20.3 National Offender Management Service (NOMS) approvals

The study will be approved by NOMS in conjunction with the NHS REC procedures. It is a requirement of NOMS that all research involving participants under NPS and CRC supervision is approved through this process.

21 INDEMNITY AND INSURANCE

The University of Plymouth (as research sponsor) and its research collaborators will be required under the terms of their collaboration agreement to maintain public liability, professional indemnity and employer's liability insurance (together with such other insurance as the sponsor may require from time to time) to cover liabilities arising from the study.
In addition, each party is required under their collaboration agreement to indemnify the other parties and their staff against all claims, proceedings, liabilities, losses and costs incurred by them as a result of or in connection with the indemnifying party's negligent acts or omissions, negligent delivery of its work under the study, negligent performance or breach of its obligations under the agreement, wilful misconduct or breach of statutory duty (including liability for damage to property, injury or death caused by any such negligent act, omission or wilful misconduct).

22 PUBLICATION POLICY

A publication plan will be developed outlining any publications and manuscripts that will be developed for peer reviewed journals. The development work may also be presented at national and international conferences.

23 FINANCE

The STRENGTHEN study is funded by the NIHR Public Health Research programme (14/54/19)

24 REFERENCES

1. Sirdifield C. The prevalence of mental health disorders amongst offenders on probation: a literature review. *J Ment Health*. 2012;21(5):485-498. doi:10.3109/09638237.2012.664305.
2. Department of Health. *Improving Health, Supporting Justice*.; 2009.
3. Hertfordshire Probation Trust. *A Health Needs Assessment of the Hertfordshire Probation Trust Caseload*. Welwyn Garden City; 2011.
4. Howerton A, Byng R, Campbell J, Hess D, Owens C, Aitken P. Understanding help seeking behaviour among male offenders: qualitative interview study. *BMJ*. 2007;334(7588):303. doi:10.1136/bmj.39059.594444.AE.
5. Light M, Grant E, Hopkins K. *Gender Differences in Substance Misuse and Mental Health amongst Prisoners: Results from the Surveying Prisoner Crime Reduction (SPCR) Longitudinal Cohort Study of Prisoners*.; 2013.
6. Department of Health. *Public Health Functions to Be Exercised by the NHS Commissioning Board: Service Specification No.29: Public Health Services for People in Prison or Other Places of Detention, Including Those Held in the Young People's Secure Estate*.; 2012.
7. Byng R, Quinn C, Sheaff R, et al. *COCOA: Care for Offenders, Continuity of Access*.; 2012.
8. Department of Health. *Choosing Health: Making Healthy Choices Easier*.; 2004.
9. Michie S, Rumsey N, Fussell S, et al. *Improving Health - Changing Behaviour: NHS Health Trainer Handbook*. London: Department of Health and British Psychological Society; 2008.
10. Attree P, Clayton S, Karunanithi S, Nayak S, Popay J, Read D. NHS health trainers: a review of emerging evaluation evidence. *Crit Public Health*. 2012;22(1):25-38. doi:10.1080/09581596.2010.549207.
11. Dooris M, McArt D, Hurley MA, Baybutt M. Probation as a setting for building well-being through integrated service provision: evaluating an Offender Health Trainer service. *Perspect Public Health*. 2013;133(4):199-206. doi:10.1177/1757913913486036.
12. Newbury-Birch D, Bland M, Cassidy P, et al. Screening and brief interventions for hazardous and harmful alcohol use in probation services: a cluster randomised controlled trial protocol. *BMC Public Health*. 2009;9(1):418. doi:10.1186/1471-2458-9-418.
13. Coulton S, Newbury-Birch D, Cassidy P, et al. Screening for alcohol use in criminal justice settings: an exploratory study. *Alcohol Alcohol*. 2012;47(4):423-427. doi:10.1093/alcalc/ags048.
14. Orr KS, McAuley A, Graham L, McCoard S. Applying an Alcohol Brief Intervention (ABI) model to the community justice setting: Learning from a pilot project. *Criminol Crim Justice*. November 2013:1748895813509636 - . doi:10.1177/1748895813509636.
15. Newbury-Birch D, Coulton S, Bland M, et al. Alcohol screening and brief interventions for offenders in the probation setting (SIPS trial): a pragmatic multicentre cluster randomised controlled trial. *Alcohol Alcohol*. July 2014. doi:10.1093/alcalc/agu046 <http://dx.doi.org/10.1093/alcalc/agu046>.
16. Kouyoumdjian FG, McIsaac KE, Liauw J, et al. A systematic review of randomized controlled trials of interventions to improve the health of persons during imprisonment and in the year after release. *Am J Public Health*. 2015;105(4):e13-e33. doi:10.2105/AJPH.2014.302498.
17. Lang N, Hillas A, Mensah M, Ryan S, Glass L. Linking probation clients with mainstream health services: Experience in an outer London borough. *Probat J*. 2014;61(3):278-285. doi:10.1177/0264550514536757.
18. Foresight Projects. *Mental Capital and Wellbeing: Final Project Report*. London; 2008. https://www.gov.uk/government/publications/mental-capital-and-wellbeing-making-the-most-of-ourselves-in-the-21st-century.
19. Ryan RM, Deci EL. Self-determination theory and the facilitation of intrinsic motivation, social development, and well-being. *Am Psychol*. 2000;55(1):68-78. http://www.ncbi.nlm.nih.gov/entrez/query.fcgi?cmd=Retrieve&db=PubMed&dopt=Citation&list_uids=11392867.
20. Bartram DJ, Yadegarfar G, Sinclair JMA, Baldwin DS. Validation of the Warwick-Edinburgh Mental Well-being Scale (WEMWBS) as an overall indicator of population mental health and well-being in the UK veterinary profession. *Vet J*. 2011;187(3):397-398. doi:10.1016/j.tvjl.2010.02.010.
21. Bartram DJ, Sinclair JM, Baldwin DS. Further validation of the Warwick-Edinburgh Mental Well-being Scale (WEMWBS) in the UK veterinary profession: Rasch analysis. *Qual Life Res*. 2013;22(2):379-391. doi:10.1007/s11136-012-0144-4.
22. Health and Social Care Information Centre. *Health Survey for England 2013*.; 2014.

http://www.hscic.gov.uk/searchcatalogue?productid=16571&q=title:“Health+Survey+for+England”&sort=Relevance&size=10&page=1#top.
23. The Scottish Government. *Scottish Health Survey 2013*.; 2014. http://www.scotland.gov.uk/Topics/Statistics/Browse/Health/scottish-health-survey/Publications#a1.
24. Stranges S, Samaraweera PC, Taggart F, Kandala N-B, Stewart-Brown S. Major health-related behaviours and mental well-being in the general population: the Health Survey for England. *BMJ Open*. 2014;4(9):e005878. doi:10.1136/bmjopen-2014-005878.
25. Taylor AH, Thompson TP, Greaves CJ, et al. A pilot randomised trial to assess the methods and procedures for evaluating the clinical effectiveness and cost-effectiveness of Exercise Assisted Reduction then Stop (EARS) among disadvantaged smokers. *Health Technol Assess*. 2014;18(4):1-324. http://www.ncbi.nlm.nih.gov/pubmed/24433837.
26. Craig P, Dieppe P, Macintyre S, et al. Developing and evaluating complex interventions: the new Medical Research Council guidance. *BMJ*. 2008;337:a1655. doi:10.1136/bmj.a1655.
27. Tennant R, Hiller L, Fishwick R, et al. The Warwick-Edinburgh Mental Well-being Scale (WEMWBS): development and UK validation. *Health Qual Life Outcomes*. 2007;5(1):63. doi:10.1186/1477-7525-5-63.
28. Maheswaran H, Weich S, Powell J, Stewart-Brown S. Evaluating the responsiveness of the Warwick Edinburgh Mental Well-Being Scale (WEMWBS): group and individual level analysis. *Health Qual Life Outcomes*. 2012;10(1):156. doi:10.1186/1477-7525-10-156.
29. Fagerstrom K. Determinants of tobacco use and renaming the FTND to the Fagerstrom Test for Cigarette Dependence. *Nicotine Tob Res*. 2012;14(1):75-78. doi:ntr137 [pii]10.1093/ntr/ntr137.
30. Reinert DF, Allen JP. The Alcohol Use Disorders Identification Test (AUDIT): a review of recent research. *Alcohol Clin Exp Res*. 2002;26(2):272-279. http://www.ncbi.nlm.nih.gov/entrez/query.fcgi?cmd=Retrieve&db=PubMed&dopt=Citation&list_uids=11964568.
31. Roe L, Strong C, Whiteside C, Neil A, Mant D. Dietary intervention in primary care: validity of the DINE method for diet assessment. *Fam Pract*. 1994;11(4):375-381. http://www.ncbi.nlm.nih.gov/pubmed/7895964. Accessed December 11, 2014.
32. Sallis JF, Haskell WL, Wood PD, et al. Physical activity assessment methodology in the Five-City Project. *Am J Epidemiol*. 1985;121(1):91-106. http://www.ncbi.nlm.nih.gov/pubmed/3964995. Accessed December 11, 2014.
33. Marsden J, Farrell M, Bradbury C, et al. Development of the Treatment Outcomes Profile. *Addiction*. 2008;103(9):1450-1460. doi:10.1111/j.1360-0443.2008.02284.x.
34. Herdman M, Gudex C, Lloyd A, et al. Development and preliminary testing of the new five-level version of EQ-5D (EQ-5D-5L). *Qual Life Res*. 2011;20(10):1727-1736. doi:10.1007/s11136-011-9903-x.
35. van Hout B, Janssen MF, Feng Y-S, et al. Interim scoring for the EQ-5D-5L: mapping the EQ-5D-5L to EQ-5D-3L value sets. *Value Health*. 2012;15(5):708-715. doi:10.1016/j.jval.2012.02.008.
36. Ware JEJ, Sherbourne CD. The MOS 36-ltem Short-Form Health Survey (SF-36): I. Conceptual Framework and Item Selection. *Med Care*. 1992;30(6):473-483. doi:10.1097/00005650-199206000-00002.
37. Brazier J, Roberts J, Deverill M. The estimation of a preference-based measure of health from the SF-36. *J Health Econ*. 2002;21(2):271-292. doi:10.1016/S0167-6296(01)00130-8.
38. Byng R, et al. *Developing and Evaluating a Collaborative Care Intervention for Prisoners, with Common Mental Health Problems, near to and after Release (Engager 2)*. Project ID: RP-PG-1210-12011; 2013.
39. New Economics Foundation. *Five Ways to Wellbeing: New Applications, New Ways of Thinking*.; 2010.
40. Gowers A, Hopkinson D, Fidan E. *Health Trainers DCRS: National Hub Report*.; 2011.
41. Markland D, Ryan RM, Tobin VJ, Rollnick S. Motivational Interviewing and Self Determination Theory. *J Soc Clin Psychol*. 2005;24:811-831.
42. Nutt DJ, Rehm J. Doing it by numbers: a simple approach to reducing the harms of alcohol. *J Psychopharmacol*. 2014;28(1):3-7. doi:10.1177/0269881113512038.
43. Lindson N, Aveyard P, Ingram JT, et al. Rapid reduction versus abrupt quitting for smokers who want to stop soon: a randomised controlled non-inferiority trial. *Trials*. 2009;10:69. doi:1745-6215-10-69 [pii]10.1186/1745-6215-10-69.
44. Ritchie JL, Spencer L. Qualitative data analysis for applied policy research. In: Bryman A, Burgess R, eds. *Analyzing Qualitative Data*. London: Routledge; 1994:173-194.
45. Teare MD, Dimairo M, Shephard N, Hayman A, Whitehead A, Walters SJ. Sample size requirements

to estimate key design parameters from external pilot randomised controlled trials: a simulation study. *Trials*. 2014;15:264. doi:10.1186/1745-6215-15-264.
46. Byng R, et al. *Engaging Offenders with Common Mental Health Problems*.; 2011.
47. Thabane L, Ma J, Chu R, et al. A tutorial on pilot studies: the what, why and how. *BMC Med Res Methodol*. 2010;10:1. doi:10.1186/1471-2288-10-1.
48. Dolgin E. Publication checklist proposed to boost rigor of pilot trials. *Nat Med*. 2013;19(7):795-796. doi:10.1038/nm0713-795.
49. *Development of a Reporting Guideline for Pilot and Feasibility Studies*. http://www.equator-network.org/library/reporting-guidelines-under-development/#25.
50. Calvert M, Blazeby J, Altman DG, Revicki DA, Moher D, Brundage MD. Reporting of patient-reported outcomes in randomized trials: the CONSORT PRO extension. *JAMA*. 2013;309(8):814-822. doi:10.1001/jama.2013.879.
51. Beecham J, Knapp M. Costing Psychiatric Interventions. In: Thornicroft G, ed. *Measuring Mental Health Needs*. 2nd ed. London: Gaskell; 2001:200-224.
52. Centre for Reviews and Dissemination. *Systematic Reviews: CRD's Guidance for Undertaking Reviews in Health Care*. York; 2009.
53. Drummond MF. *Methods for the Economic Evaluation of Health Care Programmes*. 3rd ed. Oxford ; New York: Oxford University Press; 2005. http://www.loc.gov/catdir/enhancements/fy0640/2005279427-d.html.
54. DoH. Research governance framework for health and social care (Second edition, 2005). *Health Soc Care Community*. 2005;10(1):1-54.

25 APPENDICES

Appendix 1: Descriptions of outcome measures

WEMWEBS

WEMWBS is a 14-item scale of mental wellbeing covering subjective wellbeing and psychological functioning, in which all items are worded positively and address aspects of positive mental health. The scale is scored by summing responses to each item answered on a 1 to 5 Likert scale. The minimum scale score is 14 and the maximum is 70.

Treatment Outcomes Profile (TOP)

The TOP is a tool designed to measure change and progress in key areas of the lives of people being treated in drug and alcohol services. In addition to actual drug and alcohol use over the preceding four weeks, the measures captures information on risk-taking behaviour, criminal activity, and health and social functioning. However, much of this information is being collected in other outcome measures and therefore only the section relating to drug and alcohol use is being used in the current study. However, we have expanded on the list of substances to include 'legal highs' and other substances known to be commonly used.

The total number of days abstinent over the preceding four weeks is the dependent variable. A modified version of this will be used to avoid repetition and collection of sensitive and irrelevant information.

SF-36

The SF-36 is a 36-item scale constructed to survey health status and quality of life. The SF-36 assesses eight health concepts: limitations in physical activities because of health problems; limitations in social activities because of physical or emotional problems; limitations in usual role activities because of physical health problems); bodily pain; general mental health (psychological distress and well-being); limitations in usual role activities because of emotional problems; vitality (energy and fatigue); and general health perceptions. The standard form of the instruments asks for participants to reply to questions according to how they have felt over the previous week. The items use Likert-type scales, some with 5 or 6 points and others with 2 or 3 points. Sample items include "How much bodily pain have you had during the past 4 weeks?", and "How much of the time during the past 4 weeks have you felt so down in the dumps nothing could cheer you up?" The SF-36 has been widely used and has excellent psychometrics. Further psychometric evaluation of the SF-36 has produced two summary scores: the Mental Health Component Score and the Physical Health Component Score.

EQ-5D-5L

The EQ-5D is a standardised measure of health status designed to provide a measure of health for clinical and economic appraisal. The scale comprised 5 items, each containing 5 statements indicating different degrees of health problem (e.g. no pain, slight pain, moderate pain, severe pain, extreme pain). Participants are required to tick which statement best describes their health on that day.

DINE

The DINE is a food frequency questionnaire of 19 groups of food that account for around 70% of the fat and fibre in the typical UK diet according to the National Food Survey. Each group of foods is assigned a score proportional to the fat or fibre content of a standard portion size. The scores are weighted according to the frequency of consumption. The individual scores are added together to produce total scores for fat and fibre which can then be categorised into low (a score of 30 or less), medium or high intake (score greater than 40). Completion time for an experienced interviewer is 5–10 minutes. It is free to use for clinical or research purposes. It has been designed for use by those without any nutritional knowledge.

AUDIT (AUDIT-C)

The AUDIT was developed by the World Health Organization to identify persons whose alcohol consumption has become hazardous or harmful to their health. The AUDIT is a 10-item screening

questionnaire with 3 questions on the amount and frequency of drinking, 3 questions on alcohol dependence, and 4 on problems caused by alcohol.

The AUDIT-C is a shortened version of the above using the first 3 questions only. Using a cutoff of ≥4 the Audit-C has a sensitivity of 86% of patients with heavy drinking and/or active alcohol abuse or dependence with a specificity of 72%. Using a cutoff of ≥3, AUDIT-C identifies 90% of patients with active alcohol abuse or dependence and 98% of patients with heavy drinking (specificity was only 60%, false-positive rate 40%).

It is recommended a score of ≥3 or more points on the AUDIT-C, or a report of drinking 6 or more drinks on one occasion ever in the last year, should lead to a more detailed assessment of drinking and related problems (i.e. completion of the full questionnaire).

7-Day Physical Activity Recall

Originally developed for use in the Stanford Five-City Project in the early 1980s, the PAR is a semi-structured interview that estimates an individual's time spent in physical activity, strength, and flexibility activities for the 7 days prior to the interview. The general interview format is as follows: An interviewer asks the participant to recall time spent sleeping and doing physical activities for the past 7 days. The interviewer guides the participant through the recall process, day-by-day, to determine duration and intensity of the physical activities.

Self-reported Cigarettes smoked

A self-reported number of cigarettes smoked per day, either consisting of a total number of manufactured cigarettes smoked or a number derived from the weight of rolling tobacco used daily (in grams) divided by 0.45.

Fagerström Test for Cigarette Dependence

The Fagerström Test for Nicotine Dependence is a standard instrument for assessing the intensity of physical addiction to nicotine. The test was designed to provide an ordinal measure of nicotine dependence related to cigarette smoking. It contains six items that evaluate the quantity of cigarette consumption, the compulsion to use, and dependence.

In scoring the Fagerström Test for Nicotine Dependence, yes/no items are scored from 0 to 1 and multiple-choice items are scored from 0 to 3. The items are summed to yield a total score of 0-10. The higher the total Fagerström score, the more intense is the patient's physical dependence on nicotine.

Confidence, importance, access to social support, action-planning and self-monitoring measures relating to health behaviours

Questions developed for this trial designed to reflect processes of change related to self-determined behaviour. Three questions answered on a 9-point Likert scale assess perceived importance, confidence, and access to social support associated with changing (or maintaining change) in one of the four health behaviours (smoking, diet, physical activity, and alcohol consumption), followed by questions answered on a 5-point Likert scale relating to action-planning and self-monitoring behaviour relating to the four health behaviours. These questions will only be asked to those to whom they are applicable (i.e. a non-smoker will not be asked about smoking).

Resource Use Questionnaire

This questionnaire captures participants self-reported use of various services over the past three months, including: primary care and community based services, hospital stays and outpatient appointments, visits to accident and emergency, medication use, use of services relating to education and training, use of other services (such as Probation services and legal services), and any other personal care support provided by personal carers such as friends or family. It consists of ten questions which participants answer by selecting one of the available options. The data from this is used to inform the economic evaluation.

Appendix 2 – Members of the Trial Steering Committee

Prof Sarah Stewart Brown, Professor in Public Health, University of Warwick
Dr Emma Plugge, Senior Clinical Research Fellow, University of Oxford
Prof Richard Morris, Professor in Medical Statistics, University of Bristol
Service user representatives from the Revolving Doors Agency National Service User Forum (Details to be confirmed)
Further membership TBC

Appendix 3 – Member of the Data Monitoring Committee

TBC
