# Supplementary material for: Health trainer-led motivational intervention plus usual care for people under community supervision compared with usual care alone: a study protocol for a parallel-group pilot randomised controlled trial (STRENGTHEN)
Source: BMJ Open. 2018 Jun 4;8(6):e023123. doi: 10.1136/bmjopen-2018-023123 (PMC5988189; doi:10.1136/bmjopen-2018-023123)
Supplement: Reviewer comments [file bmjopen-2018-023123.reviewer_comments.pdf]

PEER REVIEW HISTORY

BMJ Open publishes all reviews undertaken for accepted manuscripts. Reviewers are asked to complete a checklist review form (**http://bmjopen.bmj.com/site/about/resources/checklist.pdf**) and are provided with free text boxes to elaborate on their assessment. These free text comments are reproduced below.

ARTICLE DETAILS

**TITLE (PROVISIONAL)**	A Health Trainer led motivational intervention plus usual care for people under community supervision compared to usual care alone: a study protocol for a parallel group pilot randomised controlled trial (STRENGTHEN).
**AUTHORS**	Thompson, T; Callaghan, Lynne; Hazeldine, Emma; Quinn, Cath; Walker, Samantha; Byng, Richard; Wallace, Gary; Creanor, Siobhan; Green, Colin; Hawton, Annie; Annison, Jill; Sinclair, Julia; Senior, Jane; Taylor, Adrian

This article was not externally reviewed at BMJ Open. Protocols that have been independently assessed prior to submission to BMJ Open are usually fast-tracked to publication on the grounds that further substantial changes will not be possible. This independent assessment will usually be external, independent review for both a substantial grant award from a non-commercial or government funder and ethics approval.
